# Chondrogenic differentiation of mesenchymal stem cells through cartilage matrix-inspired surface coatings

**DOI:** 10.3389/fbioe.2022.991855

**Published:** 2022-09-29

**Authors:** Mingyan Zhao, Xiang Gao, Jinsong Wei, Chenlin Tu, Hong Zheng, Kaipeng Jing, Jiaqi Chu, Wei Ye, Thomas Groth

**Affiliations:** ^1^ Stem Cell Research and Cellular Therapy Center, Affiliated Hospital of Guangdong Medical University, Zhanjiang, China; ^2^ Department of Spinal Surgery, Affiliated Hospital of Guangdong Medical University, Zhanjiang, China; ^3^ Key Laboratory of Prevention and Management of Chronic Kidney Disease of Zhanjiang City, Institute of Nephrology, Affiliated Hospital of Guangdong Medical University, Zhanjiang, China; ^4^ Department of Obstetrics and Gynecology, Affiliated Hospital of Guangdong Medical University, Zhanjiang, China; ^5^ Department Biomedical Materials, Institute of Pharmacy, Martin Luther University Halle Wittenberg, Halle (Saale), Germany

**Keywords:** biomimetic multilayers, chondrogenesis, microenvironment, ERK/Sox9 pathway, noncanonical TGF-β pathway

## Abstract

The stem cell niche comprises soluble molecules and extracellular matrix components which provide chemical and mechanical cues that determine the differentiation of stem cells. Here, the effect of polyelectrolyte multilayer (PEM) composition and terminal layer fabricated with layer-by-layer technique (LBL) pairing either hyaluronan [in its native (nHA) and oxidized form (oHA)] or chondroitin sulfate (CS) with type I collagen (Col I) is investigated on chondrogenic differentiation of human umbilical mesenchymal stem cells (hUC-MSCs). Physical studies performed to investigate the establishment and structure of the surface coatings show that PEM composed of HA and Col I show a dominance of nHA or oHA with considerably lesser organization of Col I fibrils. In contrast, distinguished fibrilized Col I is found in nCS-containing PEM. Generally, Col I-terminated PEM promote the adhesion, migration, and growth of hUC-MSCs more than GAG-terminated surfaces due to the presence of fibrillar Col I but show a lower degree of differentiation towards the chondrogenic lineage. Notably, the Col I/nHA PEM not only supports adhesion and growth of hUC-MSCs but also significantly promotes cartilage-associated gene and protein expression as found by histochemical and molecular biology studies, which is not seen on the Col I/oHA PEM. This is related to ligation of HA to the cell receptor CD44 followed by activation of ERK/Sox9 and noncanonical TGF-β signaling-p38 pathways that depends on the molecular weight of HA as found by immune histochemical and western blotting. Hence, surface coatings on scaffolds and other implants by PEM composed of nHA and Col I may be useful for programming MSC towards cartilage regeneration.

## Graphical Abstract

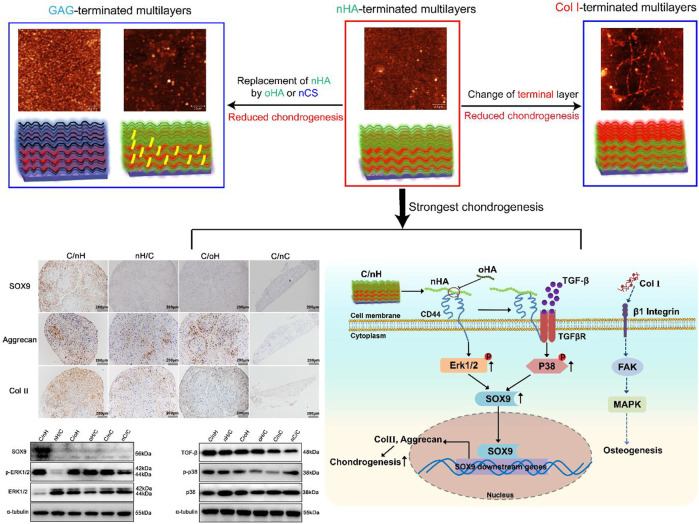

## 1 Introduction

Cartilage generally does not mend after physical trauma or degenerative diseases (e.g., osteoarthritis), because of its aneural, avascular, and alymphatic nature, as well as the limited ability of cells to migrate in the tissue for repair (Sophia Fox et al., 2009; Medvedeva EAG et al., 2018). Current techniques for treating damaged cartilage primarily include surgical interventions (Kwon et al., 2019), pharmacological therapies (Hellio Le Graverand-Gastineau, 2009), and cell-based therapies (Madeira et al., 2015). Among them, tissue engineering, which combines cells, biomaterials, and biochemical/physical factors, has been regarded as a promising approach for addressing the unsolved issue of cartilage regeneration (Vinatier et al., 2009). Mesenchymal stem cells (MSCs) are one of the dominant cell sources for tissue engineering and have been frequently used for repairing cartilage defects and injuries (Goldberg et al., 2017). Particularly, umbilical cord-derived MSC (UC-MSC) has emerged as one of the most popular transplant cell source owing to its high proliferation capacity, less affected by aging, low immune rejection response, and noninvasively harvesting method (Jo OSK et al., 2008; Balasubramanian PV et al., 2012). However, due to the multipotency of MSCs (Visweswaran et al., 2015), a specific microenvironment that can provide lineage-specific biophysical and biochemical cues for controlling the chondrogenic differentiation of MSCs for subsequent cartilage matrix production is required.

*In vivo*, MSCs reside in a niche composed of extracellular matrix (ECM), cellular (e.g., adjacent cells and their secreted factors) and mechanical components (Yin Li et al., 2015). The microenvironment regulates the fate of MSCs *via* biophysical stimulation and biochemical induction (Kaivosoja et al., 2012), which is critical for tissue morphogenesis and regeneration (Gjorevski and Nelson, 2009). The composition of the matrix has been shown to regulate cell-matrix interactions by changing the expression profile of cell adhesion molecules and the organization of the cytoskeleton, thus guiding MSC differentiation into specific lineages (Yin Li et al., 2015; Zhao et al., 2016). The physical properties of the niche are also found to influence integrin ligation and formation of focal adhesions, both of which regulate MSC differentiation (Mathieu and Loboa, 2012). The topography and mechanical properties of the ECM have an effect on the interaction between mechanosensors and mechanotransducers in cells, changing cell spreading, orientation, and gene expression, hence guiding the differentiation of stem cells (Tay et al., 2010; Abagnale et al., 2017). For example, it has been shown that MSCs grown on a softer matrix have a stronger chondrogenic potential, whereas those grown on a stiffer substrata have a greater osteogenic potential (Steward and Kelly, 2015). Indeed, the tissue-specific extracellular niche is pivotal for the optimal function of a specific cell type (Li et al., 2020), with biochemical cues, such as cartilage-specific matrix proteins, glycosaminoglycans (GAGs), and growth factors tailored into scaffold materials or hydrogels showing great potential for improving cell-ECM interactions and facilitating chondrogenic differentiation and cartilage formation (Ragetly et al., 2010; Levinson et al., 2019; Antich et al., 2020). Thus, simulating the niche surrounding cells *in vivo* is a viable approach for controlling the lineage specification of MSCs.

The native articular cartilage niche is characterized by a specialized ECM composed primarily of collagens that form a network with proteoglycans and a sparse distribution of chondroprogenitors and chondrocytes (Sophia Fox et al., 2009). Collagens are the most abundant proteins in mature articular cartilage, which are critical to the organization of the ECM and function to resist compressive forces (Guo et al., 2017). Type II collagen (Col II) is the most abundant collagen in the ECM in hyaline cartilage, forming fibrils and fibers intertwined with proteoglycan aggregates, which is regarded as a candidate biomaterial for articular cartilage regeneration (Pieper et al., 2002; Mneimneh and Mehanna, 2021). Nevertheless, type I collagen (Col I) was also found to maintain chondrocyte phenotypes and promote cartilaginous matrix production by chondrocytes and MSCs (Ohno et al., 2004; Hoyer et al., 2014; Yang et al., 2018). It is interesting to note that no significant difference in promoting effect was found between scaffold materials made from Col I and Col II (Ohno et al., 2004). Proteoglycans are the predominant non-collagenous proteins present in articular cartilage. The most abundant is aggrecan, which is decorated with chains of chondroitin sulfate (CS) and keratan sulfate, and then binds to hyaluronan (HA), forming large aggregated proteoglycans (Guo et al., 2017). HA can also interact with other cartilage proteoglycans mediated by the HA-binding region of the core protein of the interactive proteoglycans (Hascall, 1977) and bioactive proteins, such as the type IV collagen and fibrin precursor fibrinogen (LeBoeuf et al., 1986; Knudson and Knudson, 1993), which provides assembling stability to the ECM and exerts a critical role in maintaining articular chondrocyte morphology and proliferation (Allemann et al., 2001; Snyder et al., 2014). Immobilization of HA on the surface of biomaterials forms a two-dimensional (2D) HA-enriched microenvironment resembling the cartilage niche, which enhances MSCs chondrogenesis *via* the CD44/ERK/Sox9 pathway in a molecular weight (MW) dependent way (Wu et al., 2013; Wu et al., 2018a). A three-dimensional (3D) HA-enriched hybrid hydrogel with encapsulated MSCs also increased the hyaline cartilaginous matrix formation *in vitro* (Moulisová et al., 2017; Wu et al., 2018b). In addition, previous studies have shown that the presence of HA and HA-CD44 interactions can influence TGF-β1-dependent cell responses by recruiting alternative signaling pathways (Ito et al., 2004; Soma Meran et al., 2008), whereas TGF-β signaling can regulate chondrogenic differentiation of MSCs (van der Kraan et al., 2009; Wu et al., 2015; Zhang et al., 2015). As a major GAG component of native cartilage tissue, CS also possesses a remarkable bioactivity in the stimulation of proliferation and matrix production of chondrocytes and MSCs (van Susante et al., 2001; Pieper et al., 2002; Radhakrishnan et al., 2018). However, it is worth noting that CS also exists in osseous tissue units in certain amounts, where it coordinates osteoblastic cell attachment and inhibits osteoblast-mediated activation of osteoclasts through upregulating of the OPG/RANKL expression ratio, which is involved in the bone metabolic process (Pecchi et al., 2012; Kim et al., 2017). Previous studies reported a stimulatory effect of CS on the osteogenesis of MSCs related to calcium phosphate deposition and osteogenic marker gene expression, which were largely elevated when MSCs were cultured on CS-containing materials (Murphy et al., 2012; Zhao et al., 2016).

To provide a microenvironment at the biomaterial’s interface with cells and tissues, various surface functionalization techniques have been used to make them more biomimetic and thus bioactive. The layer-by-layer (LBL) technique, which is based on the alternating deposition of biogenic polyelectrolytes, such as polypeptides, proteins, and GAG for the fabrication of bioactive multilayer coatings, has the greatest potential for easy, mild, and diverse alteration of biomaterial surfaces (Zhao et al., 2014; Silva et al., 2016; Wu et al., 2016; Ren et al., 2019). Collagens combined with negatively charged GAGs, such as CS or HA, represent an interesting approach for forming multilayer coatings with a composition similar to that of native cartilage. However, no studies have been conducted to determine the effect of the molecular composition and components of the terminal layers of such biomimetic multilayers on the chondrogenic differentiation of MSCs. Specifically, the mechanism by which such biogenic multilayers regulate MSC differentiation has not been fully elucidated. In addition, intrinsic crosslinking between polyelectrolytes has recently been introduced to enhance the stability of biogenic multilayers (Zhao et al., 2014; Esmaeilzadeh et al., 2017), although the effect of intrinsic crosslinking of multilayers on the chondrogenic differentiation of MSCs has not been reported. To learn more about the effect of the matrix composition on the chondrogenic differentiation of MSCs, biomimetic multilayer systems were prepared by pairing Col I with either HA or CS as biogenic polyanions. Since HA is involved in regulation of CD44 mediated chondrogenesis (Wu et al., 2018a), we chose the following two different types of HA for this study: oxidized HA (oHA), which is characterized by lower molecular weight and the ability to form intrinsic cross-links with collagen, and native HA (nHA). In contrast, CS was only used in its native form to reduce the number of systems. To learn more about the effect of these microenvironment on chondrogenic differentiation of MSC, we characterized cell morphology and viability, hyaladherin CD44 clustering profile, and organization of cytoskeletal structures. Furthermore, the expression of major chondrogenic markers at both the gene and protein levels by RT-PCR, western blot (WB), immune and histochemical staining were investigated. We also studied the involvement of related signal pathways that control chondrogenesis of MSC. Findings of this study indicate that multilayers composed of nHA and Col I promote chondrogenic differentiation of MSCs related to an upregulated signal transduction pathways of ERK/SOX9 showing that LBL technique can be used to fabricate biomimetic ECM like environments.

## 2 Materials and methods

### 2.1 Materials

Native hyaluronan (nHA, MW∼1.3 MDa) was supplied by Innovent (Jena, Germany) while native chondroitin sulfate (nCS, MW∼25 kDa), poly (ethylene imine) (PEI, Mw∼750 kDa), mercaptoundecanoic acid (MUDA, 95%), and sodium chloride (NaCl) were supplied by Sigma (St. Louis, United States). Type I collagen (Col I) (from porcine skin, MW∼100 kDa) was supplied by Sichuan Mingrang Biotech (Sichuan, China). Sodium hydroxide (NaOH), potassium chloride (KCl), hydrochloric acid (HCl), acetic acid (HAC), ammonium hydroxide (NH_3_·H_2_O), hydrogen peroxide (H_2_O_2_), and ethanol were purchased from Roth (Karlsruhe, Germany). Phosphate buffered saline (PBS) was supplied by Gibco (Grand Island, United States). New gold coated quartz sensors were purchased from Biolin Scientific (Göteborg, Sweden). Glass coverslips were purchased from Menzel (Braunschweig, Germany) and silicon wafers were from Kaufering (Germany). Prior to use, new gold sensors were cleaned with ethanol p. a. (99.5%) (Guangdong Guanghua Chemical Factory Co., Ltd, Guangzhou, China), followed by drying with a flow of nitrogen. Thereafter, the cleaned sensors were incubated in 2 mm mercaptoundecanoic acid (MUDA, Sigma) in ethanol p. a. at room temperature overnight to obtain a negatively charged surface comparable to glass (Niepel et al., 2009). Glass coverslips were cleaned for 2 h with NaOH (0.5 M in 96% ethanol), then thoroughly rinsed with ultrapure water and dried under nitrogen flow. Silicon wafers were cleaned with a solution of 25% NH_3_·H_2_O, 35% H_2_O_2_, and ultrapure water at a volume ratio of 1:1:5 at 75°C for 15 min, followed by thoroughly rinsing with ultrapure water.

### 2.2 Formation of polyelectrolyte multilayers (PEM)

The synthesis of oxidized HA (oHA, with an oxidation degree of 38.8%) and the formation of polyelectrolyte multilayers (PEM) based on native or oxidized glycosaminoglycans (nGAG or oGAG) have been described previously (Zhao et al., 2014). nHA, oHA, and nCS polyanion solutions (each 0.5 mg ml^−1^ in 0.15 M NaCl solution, pH adjusted to 4), as well as polycation solutions of Col I (0.5 mg ml^−1^ in 0.2 M HAC supplemented with 0.15 M NaCl) and PEI (5 mg ml^−1^ in PBS, pH adjusted to 7.4) were prepared. The PEI solution was allowed to adsorb onto the substrata (either cleaned glass coverslips, silicon wafers, or cell culture plates, as specified later in each section) for 30 min, forming an anchoring base layer with a positive net charge (Zhao et al., 2014), followed by a 5-min wash in triplicate with ultrapure water. Following that, the substrata were incubated in polyanion solutions of nHA, oHA, or nCS for the first polyanion layer for 15 min, washed with 0.15 M NaCl (pH 4.0), and followed by a 20-min adsorption of the polycation Col I layer. Each adsorption step was followed by a wash with NaCl. Finally, PEM systems with eight layers (terminal layer of Col I) or seven layers (outermost layer of nHA, oHA, or nCS) on top of the PEI layer were obtained. These systems were designated as nH/C, C/nH, oH/C, C/oH, nC/C, and C/nC.

### 2.3 Study of multilayer formation with quartz crystal microbalance (QCM)

QCM measurements were performed using a QCM device with dissipation measurement (QCM-D, Q-Sense Analyzer-E4, Biolin Scientific, Göteborg, Sweden) to monitor the multilayer formation process. The adsorption time for each polyelectrolyte solution and the rinsing step were the same as described in Section 2.2. The measurements were conducted in triplicate and data were fitted using Q-Sense Dfind (version 1.2.7, Biolin Scientific). Seven different harmonics (from the first to the 13th) and their corresponding dissipation factors were recorded by the QCM-D. When applying an alternating potential, the QCM-D sensor will oscillate at its resonance frequency (*f*). Mass adsorbed at or desorbed from the sensor surface causes a shift in this frequency in real-time, which was quantified as frequency and dissipation shifts (*Δf* and *ΔD*). When the multilayer film is rigid, thin, and evenly distributed, changes in adsorbed mass (*Δm*_QCM-D_) and thickness (*Δδ*) can be related to *Δf*, which can be determined by Sauerbrey Eqs 1, 2, respectively (Sauerbrey, 1959), as given below:

$$∆m_{QCM-D}=-\frac{C}{n}∆f_{n}$$ (1)

$$∆\delta =\frac{∆f/n\times C}{100}$$ (2)

here *n* (*n* = 1, 3, 5, … , 13) represents the overtone number, *C* represents the mass sensitivity constant depends on the quartz crystal and *Δf*_*n*_ represents the frequency shift. The quartz crystals used here were as follows: *f*_*0*_ = 5 MHz and *C* = 17.7 ng/cm^−2^ Hz.

When the driving potential is switched off, the dampening of oscillatory motion is connected to the structural properties of the additional layer on the sensor surface, which can be quantified as energy dissipation (*ΔD*).

### 2.4 Surface topography of polyelectrolyte multilayers (PEM)

The surface topography of the various multilayers was viewed by atomic force microscopy (AFM) using a device from Nano-R, Pacific Nanotechnology (Santa Clara, CA). Clean and PEM-coated silicon wafers (either Col I or GAG-terminated) with a size of 10 × 10 mm^2^ were investigated in close-contact mode under ambient (air) conditions, with scans of approximately 3 × 3 μm^2^ for each sample. The software “Gwyddion v2.30” was used for data post-processing.

### 2.5 Cell culture

Mesenchymal stem cells from human umbilical cord (hUC-MSCs) were extracted and characterized as described in our previous work (Gao et al., 2022). The isolated hUC-MSCs were grown in low-glucose Dulbecco’s modified Eagle’s medium (L-DMEM, Gibco, Beijing, China) supplemented with 10% fetal bovine serum (FBS, Gibco) and 1% penicillin-streptomycin at 37°C in a humidified environment containing 5% CO_2_. When cells reached almost confluence, 0.25% trypsin/0.02% EDTA (Gibco) was used to remove them from the flasks. The harvested cells were centrifuged at 1,000 rpm for 3 min and then resuspended in 10% FBS containing L-DMEM at a predetermined concentration (described later in each section) and seeded onto the various PEM.

### 2.6 Cell adhesion investigation

Before cell seeding, the plain or PEM-coated glass coverslips were sterilized by UV irradiation (for 30 min on each side) and then placed in 24-well tissue culture plates (NEST, Wuxi, China). Serum-free suspensions of hUC-MSCs at a concentration of 10,000 cells mL^−1^ were prepared and seeded on plain or PEM-coated samples. Cellular adhesion complexes, such as CD44, and cellular structures, such as filamentous actin and nuclei, were visualized using immunofluorescence and fluorescence stains with confocal laser scanning microscopy (CLSM). After incubation, cells were fixed with 4% paraformaldehyde solution (RotiHistofix, Sigma) and permeabilized with 0.1% Triton X-100 in PBS (v/v) (Sigma) (each for 10 min). After rinsing the samples twice with PBS, they were blocked by incubation for 1 h with bovine serum albumin (BSA, 1% (w/v) in PBS, Sigma), followed by washing with PBS. Hyaladherin CD44 was stained with a monoclonal anti-CD44 antibody (1:200, CST, Danfoss, Massachusetts, United States) at room temperature for 1 h followed by AlexaFluor 488 goat anti-mouse (1:400, CST) as a secondary antibody. Simultaneously, actin was visualized by incubating with phalloidin-Alexa Fluor 568 (1:50, Life, Eugene, OR, United States) for 1 h. Nuclei were stained and mounted with DAPI (Vector Laboratories, Inc, Burlingame, CA) after rinsing with PBS and distilled water. Finally, the samples were examined and photographed using CLSM (Olympus FV300, Tokyo, Japan). Images were processed with the Olympas FV31S-SW. To quantify the expression levels of CD44 in cells, images were analyzed by ImageJ according to a reported method with slight modification (Ochsner MT et al., 2010). An outline was drawn around each cell and area using the freehand selection tool, and the area, mean, and integrated intensity were determined. Next, using a rectangular selection tool, a region next to the cell of interest was selected as the background signal, and the area, mean, and integrated density were calculated in the same way. Finally, the total corrected cellular fluorescence (*CTCF*) was calculated following Eq. 3:

CTCF=ID−(AC×MFBR) (3)

Here *ID* represents the integrated intensity, while *AC* is the area of selected cells and *MFBR* is the mean fluorescence of background readings.

### 2.7 Induction of chondrogenic differentiation

Chondrogenic differentiation was initiated after a confluent monolayer was formed. The chondrogenic differentiation medium was composed of basal medium (L-DMEM containing 1% penicillin-streptomycin and 1% FBS), 20 nm dexamethasone (Sigma), 5 μg ml^−1^ ascorbic acid (Sigma), 1% ITS (v/v), and 10 ng ml^−1^ TGF-β1 (Peprotech, Rocky Hill, United States). The medium was changed every 3 days. The samples on which cells were grown with basal medium were used as a reference.

### 2.8 Detection of chondrogenic differentiation of hUC-MSCs

#### 2.8.1 Gene expression of chondrogenic markers by quantitative real-time PCR (qRT-PCR)

To evaluate the extent of differentiation of hUC-MSCs toward a chondrogenic lineage, expression levels of major chondrogenic markers, such as Sox9, Aggrecan, Col II, and osteogenic markers Col I were studied. On day 7 and day 14 post-differentiation, cells were washed once with PBS, and total RNA was extracted using RNAiso Rlus (Takara, Shiga, Japan) following the standard protocols (Chomczynski and Sacchi, 1987). Thereafter, the extracted RNA was transcribed into cDNA using PrimeScriptTM RT Master Mix Kit (Takara) based on the manufacturers’ protocol. The SYBR Premix Ex TaqTM II (Takara) was used for qRT-PCR. Reactions were conducted using a LightCycler480 Real-Time PCR System (Roche, Basel, Switzerland) according to the following protocol: 95°C for 5 min, 40 cycles of 95°C for 15 s, 60°C for 15 s, and 72°C for 30 s. The expression of mRNA in hUC-MSCs was evaluated using specific primers (see Table 1) and GAPDH as the endogenous control. Data were calculated using the 2^^−ddct^ method based on Livak (Livak and Schmittgen, 2001). The plain glass coverslips on which cells were cultured with basal medium or chondrogenic differentiation medium were used as references.

**TABLE 1:** Specific primers for qRT-PCR.

	Forward primer	Reverse primer	Size (bp)
GAPDH	CAG​ACC​ACA​GTC​CAT​GCC​ATC​AC	GAC​GCC​TGC​TTC​ACC​ACC​TTC	275
Sox9	AGG​AGA​GCG​AGG​AGG​ACA​AGT​TC	TGT​TCT​TGC​TGG​AGC​CGT​TGA​C	120
Aggrecan	TGA​CAC​ACA​CAC​CCC​AGC​TT	ATA​GGC​GGA​CGT​CTC​ACT​GC	140
Col II	CCT​GGC​AAA​GAT​GGT​GAG​ACA​G	CCT​GGT​TTT​CCA​CCT​TCA​CCT​G	149
Col I	GAT​TCC​CTG​GAC​CTA​AAG​GTG​C	AGC​CTC​TCC​ATC​TTT​GCC​AGC​A	107

### 2.8.2 Histochemical analysis

After 21 days of induction, aggregation of cells and formation of larger clusters resembling cartilage microtissue were found on PEM-coated surfaces treated with chondrogenic differentiation medium, while no microtissue formed on plain or basal medium-treated surfaces. As a result, samples were divided into the following two groups: those with no microtissues (Samples 1) were directly stained with Safranin O (Solarbio), while those with microtissues (Samples 2) were harvested and fixed in 4% formalin for 24 h. Furthermore, Samples one was rinsed once with PBS and then fixed with 4% paraformaldehyde for 10 min. Following that, the samples were rinsed twice with distilled water and stained with Safranin O solution in the dark at room temperature for 30 min. Finally, excess dye was washed away with distilled water, and the images were captured using an inverted fluorescence microscope. Samples two fixed with formalin were dehydrated using a graded series of ethanol solutions, cleaned with xylene (Tianjin Damao Chemical Reagent Factory Co., Ltd, Tianjing, China), and then embedded in paraffin. Sections were cut to a thickness of 6 µm and collected on glass slides before deparaffinization and stained with Safranin O according to a defined method for visualizing acidic GAG production. The stained sections were shown and evaluated using an Olympus BX53 microscope (Olympus) equipped with an Olympus DP74 digital camera.

#### 2.8.3 Immunohistochemical analysis

The deparaffinized and redehydrated sections were boiled in 0.01 M citrate buffer (pH 6.0, Solarbio) for 10 min, and then washed with distilled water. The immunohistochemistry staining was performed following the manufacturer’s instruction (SP Rabbit & Mouse HRP Kit (DAB), Cowin Bio, Beijing, China). Briefly, the sections were cleared with xylene, rehydrated in graded ethanol solutions, and then rinsed with 0.01 M PBS (pH 7.4). Antigen retrieval was conducted by incubating these sections in citrate buffer, heating them in a microwave oven for 15 min, and then cooling them at room temperature. After rinsing again with 0.01 M PBS, the endogenous peroxidase activity was quenched by immersing in blocking buffer, and nonspecific binding sites were blocked with normal goat serum in a moist chamber at room temperature for 30 min. Next, the sections were incubated with primary antibodies against Sox9 (1:100, ZEN-BIOSCIENCE, Chengdu, China), Col II (1:100, Abcam, Cambridge, UK) and Aggrecan (1:100, Sigma) overnight at 4°C, and then extensively rinsed with PBS. Thereafter, the sections were incubated with secondary antibodies for 60 min, washed again with PBS, and incubated with streptavidin-HRP for 60 min. Finally, the antibody binding sites were viewed using a 3,3′-diaminobenzidine tetrahydrochloride (DAB) solution, and the sections were counterstained with hematoxylin (Solarbio) and mounted with coverslips for microscopy.

#### 2.8.4 Western blot (WB) assay

To further investigate the expression levels of cartilage-related proteins and to reveal the potential signaling pathways involved in the regulation of chondrogenic differentiation of hUC-MSCs stimulated by the PEM, WB assays were performed. After induction for 21 days, cells grown on the different PEM were washed once with sterile PBS and then treated with radio immunoprecipitation assay lysis buffer (Thermo Fisher Scientific, Rockford, United States) to obtain cell lysates. To ensure sufficient lysis, cells formed into the chondrogenic microtissues were further ultrasonically disrupted using a Tissuelysey-24 (Shanghai Jing Xin, China) at 60.0 Hz for 50 s. Thereafter, centrifugation was performed at 12,000 g for 15 min on all cell lysates to remove the cell debris. Protein concentrations were determined by the BCA Protein Assay kit (Beyotime, Shanghai, China). Equal amounts of protein were run on an 8% or 10% sodium dodecyl sulfate polyacrylamide gel before transferring the gels onto a polyvinylidene fluoride membrane (Merk Millipore, Cork, Ireland). The membrane was then blocked in TBS containing 5% skimmed milk (Becton, Dickinson and Company, Sparks, United States) for 1 h, followed by incubation with primary antibodies against phosphorylated P38 (p-P38) (Santa Cruz, CA, United States), total P38 (Santa Cruz), p-ERK1/2 (Cell Signaling Technology, Massachusetts, United States), total ERK1/2 (Cell Signaling Technology), TGF-β (Abcam), Sox9, Aggrecan, Col II, and α-tubulin (Santa Cruz) at 4°C overnight. All primary antibodies were used at a dilution of 1:1,000. Next, the membrane was washed with tris-buffered saline with tween and incubated with the horseradish peroxidase (HRP)-conjugated secondary antibodies (diluted 1:5,000, Santa Cruz) at room temperature for 1 h. The antibody-reactive bands were detected with the WesternLumaxLight™ Superior kit (ZETA™ Life, Menlo Park, CA, United States) and visualized using the BG-gdsAUTO720 Gel imaging system (Baygene Biotech Company Limited, Beijing, China).

### 2.9 Statistical analysis

Results were shown as the mean ± standard deviation (SD). The differences between groups were analyzed using One-way ANOVA with Tukey’s multiple comparison test. A *p* value of<0.05 (*p < 0.05) was considered as statistically significant.

## 3 Results and discussion

### 3.1 QCM measurements

**FIGURE 1:**
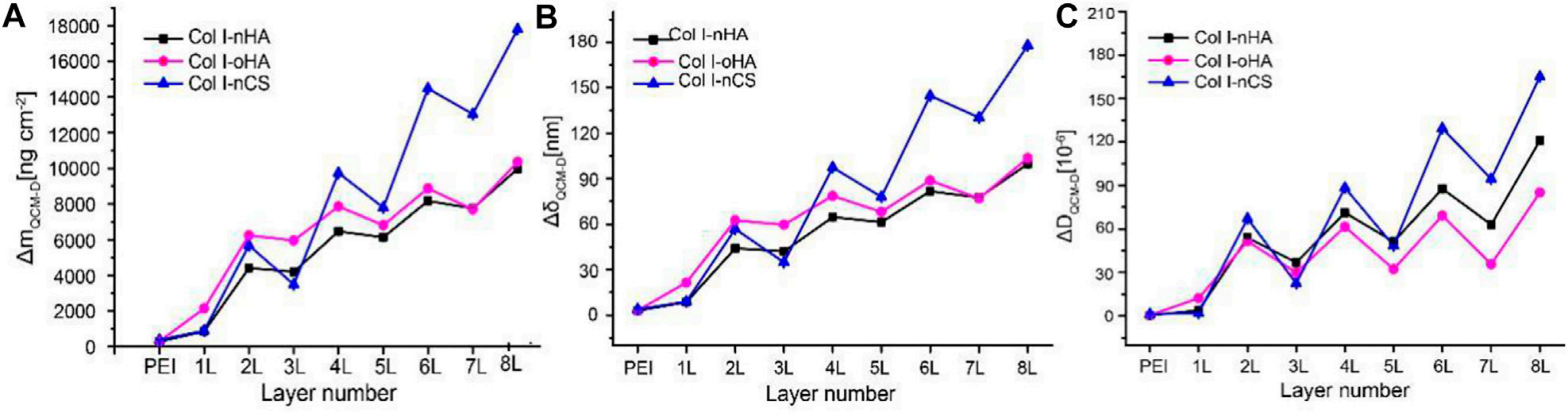
**(A,B)** The mass increase and thickness of layers detected by QCM-D using the Sauerbrey equation. **(C)** The shift of dissipation (ΔD) during multilayer formation determined by QCM-D. Odd layers: polyanion (nHA, oHA, or nCS), even layers: polycation Col I.

In order to show the establishment and structure of these PEM surface coatings, QCM and AFM were performed. The QCM-D measurements allowed the assessment of total layer mass, layer thickness, and shift in dissipation (ΔD) as presented in Figure 1. The total layer mass includes the amounts of adsorbed polyelectrolytes and solvent, while ΔD reflects the properties of the adsorbed material, where rigid and thin layers give rise to a smaller response than soft or thick and loosely attached layers. The multilayer growth of all the systems shows a staircase-shaped curve with lower mass increase by GAGs (nHA, oHA, or nCS, odd layer number) but higher by Col I (even layer number). However, unlike the steady increase in adsorption for the nCS-containing PEM, the adsorption almost reached a plateau for HA-based (both nHA and oHA) PEM, indicating that mass adsorption achieved an equilibrium. In addition, a significantly larger amount of Col I deposition was observed for nCS-based PEM, probably owing to the higher charge density of nCS compared to HA, which increased the binding of Col I. Indeed, the adsorption and fibrillization of Col I were strongly influenced by the type of GAG as it will be shown in next sections. Furthermore, it was visible that the multilayer mass and thickness of the oHA-based PEM were higher owing to additional covalent crosslinking between the aldehyde groups of oHA and amino groups of Col I. In contrast, nHA-containing PEM showed a much lower mass and thickness increase, because only ion pairing is the dominating mechanism (Zhao et al., 2014; Esmaeilzadeh et al., 2017). The staircase pattern increase during the assembly could be explained by the diffusion of GAGs into the PEM that increased ion-pairing between functional groups (e.g., carboxyl of GAGs and amino groups of Col I) displacing counter ions and water molecules decreasing mass, which resulted in almost no increase in the mass adsorption and a lower film thickness. Indeed, a stiffening of multilayers with lower hydration of PEM was also evidenced by a significant drop in dissipation values (see Figure 1C **)** upon nHA, oHA, or nCS adsorption caused by the increased ion painting inside PEM. It is of note that the stiffening of multilayers was more pronounced upon the adsorption of nCS than that of HA (both nHA and oHA). The same phenomenon has been observed in previous studies and is probably caused by the easier diffusion of nCS into the underlying layers due to its low molecular weight and high charge density, resulting in stronger ion pairing with compaction of multilayers (Lundin et al., 2011; Aggarwal et al., 2013). Nevertheless, the overall ΔD values were higher on nCS-based PEM as compared to HA-containing ones (both nHA and oHA), which is probably caused by more adsorption of Col I that increased the layer thickness and viscosity (Hüseyin Deligöz and Tieke, 2014). Figure 1C also demonstrates that the ΔD values were lower for oHA-based multilayers compared to nHA-containing ones, indicating that the additional chemical crosslinking increased the stiffness of the multilayers, which corresponds to previous findings (Wang et al., 2015).

### 3.2 Atomic force microscopy studies of multilayer surface topography

**FIGURE 2:**
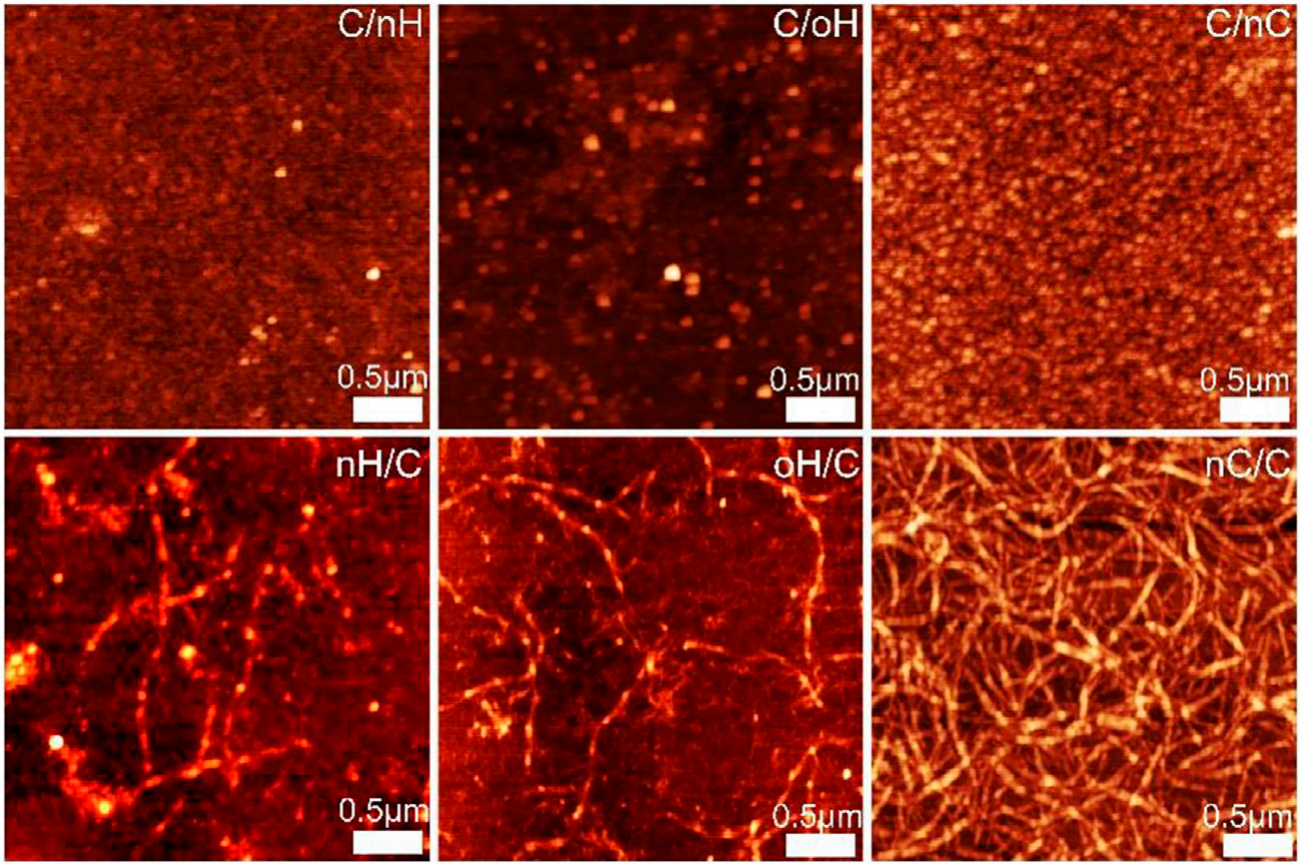
AFM images of polyanion (nHA, oHA, and nCS) terminal layers and outermost Col I for the different PEM. Scan size of all the images is 3 × 3 µm.

The surface topography of the outermost Col I and polyanion terminal layers of different PEM was investigated by AFM in a dry state. Figure 2 demonstrates that the surface topography of multilayers is highly dependent on the type of GAG as well as the terminal layer. The Col I aggregates forming fibrillar structures were found in multilayers with Col I as the terminating layer, while a granular surface structure was observed in the outermost polyanion layer (Niepel et al., 2009; Aggarwal et al., 2013). However, it is also visible that the surface morphology of the terminal polyanion layers greatly differed when different GAG was used for multilayer formation. A dense and evenly-distributed granular structure was observed for nHA-terminated multilayers, while the presence of nCS as a polyanion led to an increased granularity of these multilayers, which might be explained by the higher charge density of nCS compared to HA, which encouraged the binding of more nCS. Interestingly, the outermost oHA layer is still organized into granules, but these are larger, which could be due to aldehyde groups that form more bonds with the Col I chain than ion pairing only in nHA-based PEM. Furthermore, for all Col I-terminated PEM, it is of note that a significant fibrous structure was observed on nCS-containing multilayers, forming a network-like structure, while rather short and sparse fibrils were found in HA-based (both nHA and oHA) PEM, which formed rather discrete aggregates than fibers. Col I fibrillogenesis is a rather complex process affected not only by environmental factors, such as Col I concentration, electrolyte type, and pH value but also by the presence of polysaccharides and other naturally derived polyanions. Particularly, the presence of CS accelerated the fibril formation of collagen (Wood, 1960). In addition, previous studies have shown that the concentration of collagen also highly affects fibril formation, the higher concentration (24 μg ml^−1^) dramatically promoted collagen fibrillogenesis, while the lower concentration (6 μg ml^−1^) was not (Cisneros et al., 2006). We could show with QCM (Figure 1) and WCA (Supplementary Figure S1) investigations here that considerably less Col I was present in multilayers with HA (both nHA and oHA) as polyanion, while an apparently larger amount of Col I was deposited in nCS-based PEM. In line with this, rather elongated globules were observed in HA-based multilayers, while distinguished fibril formation was found in nCS-containing ones.

### 3.3 Initial interactions of hUC-MSCs with polyelectrolyte multilayers (PEM)

**FIGURE 3:**
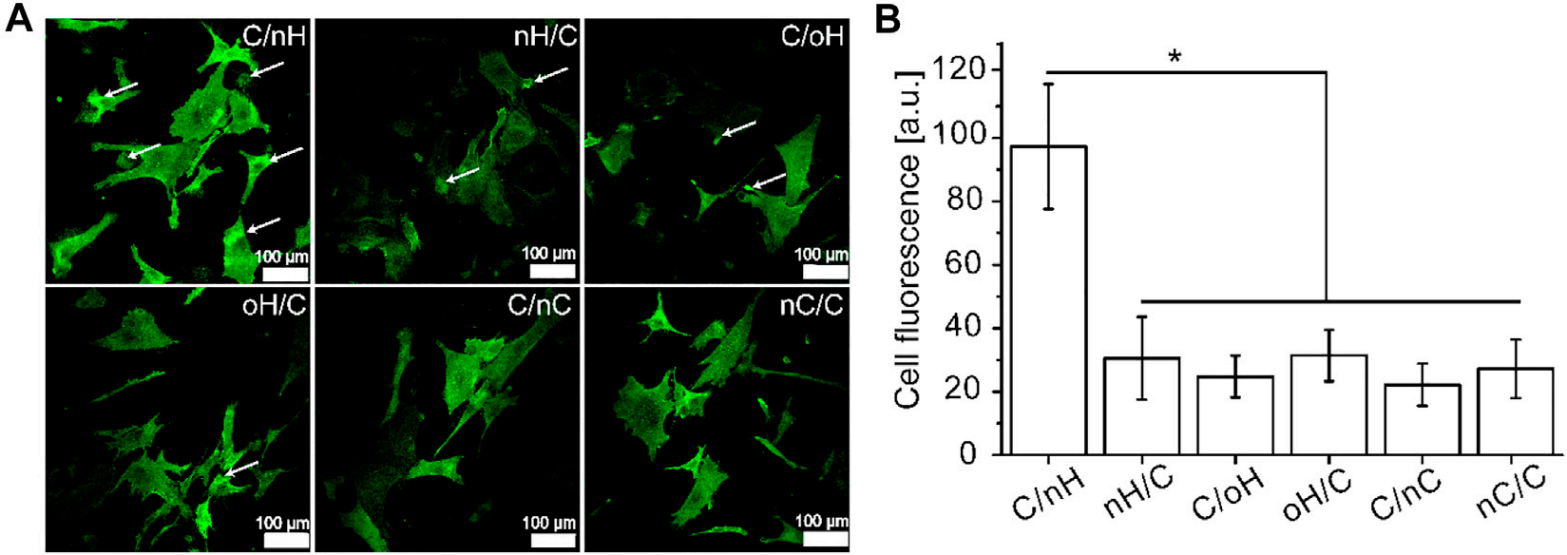
The adhesion of hUC-MSCs grown on different PEM **(A)** CLSM with staining of CD44 (green) of hUC-MSCs after 4 h incubation on the different PEM, and the fluorescence signals of CD44 **(B)** was quantified by ImageJ and corrected total cell fluorescence was calculated by fluorescence signal with elimination of background signal [PEM were prepared with terminal layer of nHA, oHA and nCS (C/nH, C/oH, C/nC) or outermost layer of Col I (nH/C, oH/C, nC/C). Cells were seeded in serum free medium for 4 h].

Since the interaction between HA and CD44 is crucial for mediating chondrogenesis of MSCs (Wu et al., 2018a), here, we investigated the CD44 clustering of hUC-MSCs on the various PEM. As shown in Figure 3A, CD44 was considerably more expressed and organized in hUC-MSCs adhering on Col I/nHA surfaces, while on nCS-based PEM, a weaker expression of CD44 with no tendency for clustering was observed. The highest expression of CD44 on Col I/nHA was further demonstrated by quantification, as shown in Figure 3B. It is also interesting to note that signs of CD44 clustering contacts with neighboring cells were visible in cells on the Col I/nHA surface. In addition, with respect to the expression of CD44, no difference was found between the HA-based PEM (both nHA and oHA) when Col I was used as the terminal layer. However, the pericellular accumulation of CD44 was considerably stronger in cells grown on the Col I/nHA surface as compared to Col I/oHA. After oxidization, the molecular weight of HA was drastically decreased from 1,300 kDa to 55 kDa (See Supplementary Table S1), which might reduce the binding between HA and its receptor, and thus, reduce the clustering of CD44 on Col I/oHA. Indeed, it has been demonstrated that the concentration of HA and its molecular weight are of great importance for the interactions between HA and the membrane receptors (Amorim et al., 2018). Due to the multitude of possible binding sites, the higher molecular weight HA can effectively crosslink several CD44 receptors, and thus, stimulate CD44 clustering, whereas lower molecular weight HA with reduced binding sites disrupts it (Yang et al., 2012; Amorim et al., 2018; Julio, 2018).

### 3.4 Chondrogenic differentiation of hUC-MSCs

#### 3.4.1 Characterization of chondrogenesis of hUC-MSCs

**FIGURE 4:**
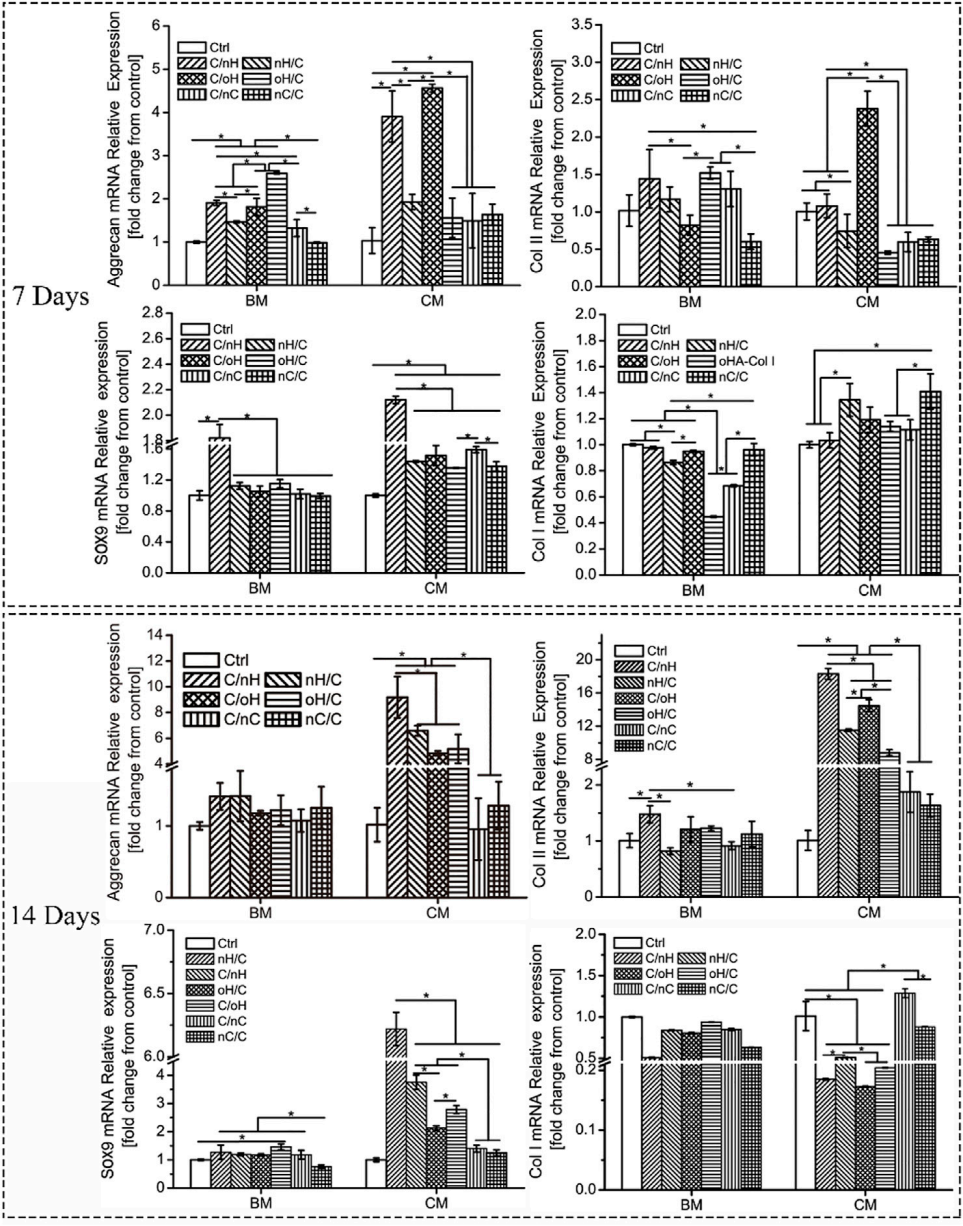
Relative expression levels of chondrogenic-associated genes in hUC-MSCs grown on different PEM at different time points. [BM: basal medium; CM: chondrogenic differentiation medium]. The multilayers are the same as described in Figure 3.

To determine the effect of molecular composition and terminal layer on the chondrogenic differentiation of hUC-MSCs, we investigated the expression of cartilage-specific genes, such as Sox9, Aggrecan, and Col II at different time points. As shown in Figure 4, even for the cells cultured in basal medium, the chondrogenesis-associated genes were upregulated on Col I/nHA, although some of them were statistically insignificant compared to the control on day 7 of differentiation. For the cells cultured in either basal medium or chondrogenic differentiation medium, the highest expression of Sox9 was found on Col I/nHA, while no large difference in the expression of Sox9 was found among the other PEM. However, the expressions of Aggrecan and Col II varied greatly among the different PEM, particularly when cells were treated with chondrogenic differentiation medium. Interestingly, the HA-containing (both nHA and oHA) multilayers showed a significant promoting effect on the expression of cartilage-specific genes compared to the nCS-based PEM. It is also of interest to note that, generally, the expression of these genes is higher in the GAG-terminal layers in comparison to the outermost Col I layer. After induction for 14 days, the expressions of chondrogenic-specific markers are still slightly higher on Col I/nHA compared to control in basal medium, and a significant difference is found with respect to the expression of Col II. For the cells treated with chondrogenic differentiation medium, the expressions of chondrogenesis-associated genes, particularly Col II, are further enhanced by the HA-containing (both nHA and oHA) multilayers when compared to the control group. Again, HA-based multilayers (both nHA and oHA) show a positive effect on increasing expression of Sox9, Aggrecan, and Col II genes compared to nCS-containing multilayers. Specifically, Col I/nHA induces the most upregulated expression of these genes, indicating that this PEM has the strongest promoting effect on enhancing the chondrogenic differentiation of hUC-MSCs. Another interesting finding is the always lower expression of the Col I gene on Col I/nHA during the observation time, indicating this PEM may induce cell differentiation into hyaline cartilage but not fibrocartilage, which is desirable for cartilage tissue engineering (Ohno et al., 2004). Col II is the major component of hyaline cartilage that is present on the articular surface of the bone, while Col I fibers are contained in fibrocartilage, which is found on the meniscus.

**FIGURE 5:**
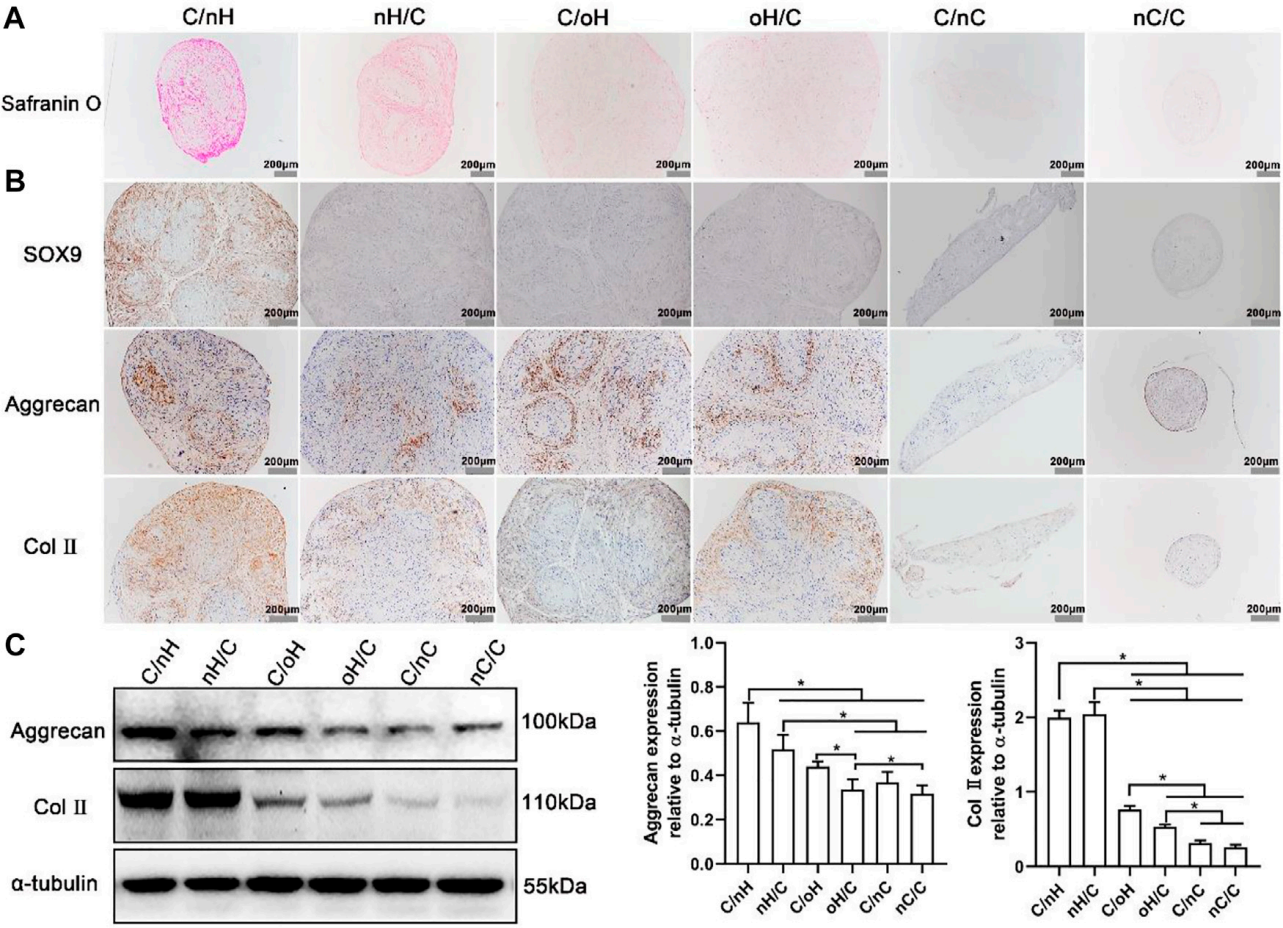
The determination of chondrogenic differentiation of hUC-MSCs placed on the different PEM **(A)** Safranin O staining for acidic glycosaminoglycans (GAGs) in hUC-MSCs placed on the different PEM treated with chondrogenic differentiation medium. After induction for 21 days, the cells formed microtissues were embedded in paraffin and sliced into sections and then stained with Safranin O; **(B)** Immunohistochemical staining images for Sox9, Aggrecan and Col II in cell microtissues formed on the different multilayer surfaces after 21 days culture in chondrogenic differentiation medium; **(C)** Representative blots showing the expression levels of chondrogenic-related proteins in hUC-MSCs placed on the different multilayer surfaces after 21 days of induction in chondrogenic differentiation medium, and the associated bar plots displaying the ratios of Aggrecan and Col II to tubulin as evaluated by densitometric analysis of western blots. The multilayers are the same as described in Figure 3.

**FIGURE 6:**
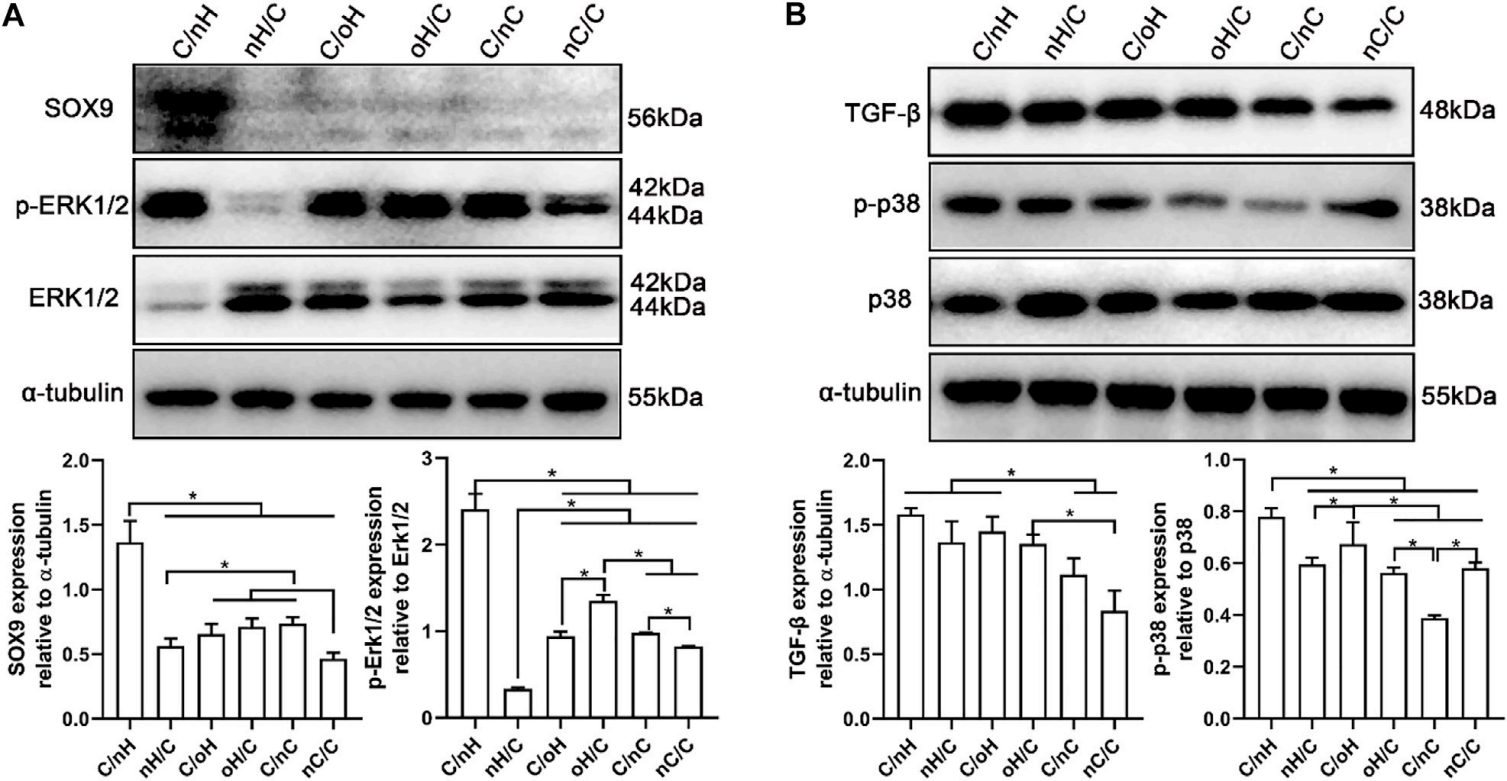
ERK/Sox9 and p38 pathways are involved in the multilayers-dependent differentiation of hUC-MSCs **(A)** Representative blots showing the expression levels of ERK/Sox9 signal pathway involved proteins in hUC-MSCs placed on the different multilayer surfaces after 21 days of induction in chondrogenic differentiation medium and the qualitative data showing the ratios of p-ERK/ERK and Sox9 to tubulin as evaluated by densitometric analysis of western blots. **(B)** Representative blots showing the expression levels of p38 signal pathway involved proteins in hUC-MSCs placed on the different multilayer surfaces after 21 days of induction in chondrogenic differentiation medium and the qualitative data showing the ratios of TGF-β and p-p38/p38 to tubulin as evaluated by densitometric analysis of western blots. Data are representative and displayed as the mean ± SD of three independent experiments. The multilayers are the same as described in Figure 3.

To confirm the cartilage matrix formation of hUC-MSCs on the different PEM, Safranin O staining was performed to visualize the acidic GAG deposition after 21 days of induction (Supplementary Figure S4; Figure 5A). As in correspondence with the PCR results, when cells are treated with basal medium, no positive staining was found on control and nCS-containing samples, while a slight staining was observed on HA-based multilayers, particularly on Col I/nHA (Supplementary Figure S4). Nevertheless, though the chondrogenesis-related genes are slightly more expressed on the Col I/nHA, only some small cell aggregates but no cartilage-like microtissues were formed on this PEM. In contrast, except for the control group, microtissues are developed on all the PEM-coated surfaces (Figure 6A) and show positive staining for Safranin O when cells are cultured in chondrogenic differentiation medium, indicating that the multilayer alone is not sufficient to support the chondrogenesis of hUC-MSCs and that chondrogenic supplementals are needed. It is also interesting to note that the intensity of staining greatly varied among the different PEM, with the strongest staining visible on Col I/nHA, suggesting the most cartilage matrix deposition. Generally, the staining of Safranin O was more intense on the HA-based systems compared to the nCS-containing ones, and the GAG-terminated systems showed more acidic GAG deposition than the Col I-terminated ones.

The expression of cartilage-specific markers at the protein level was also evaluated after 21 days of induction using immunohistochemical staining and a WB assay to further investigate the effect of molecular composition and terminal layer on the chondrogenic differentiation of hUC-MSCs. As the cells cultured in basal medium and on the plain coverslips failed to form cartilage-like microtissue and could not favor the differentiation of hUC-MSCs into chondrogenic lineage, we studied only the cells grown on PEM-coated surfaces treated with chondrogenic differentiation medium. As shown in Figure 5B, a significant staining of Aggrecan and Col II was visible on HA-based PEM, while only weak staining was detectable on the multilayer surfaces containing nCS. Again, the most intensive staining of Aggrecan and Col II was found on Col I/nHA, and the staining was always stronger on the GAG-terminal layers in comparison to the Col I-terminated ones. Further, as detected by the WB assay shown in Figure 5C, the cartilage-associated proteins, such as Aggrecan and Col II, are also considerably more expressed in cells cultured on HA, particularly on the nHA-based multilayers compared to the nCS-containing ones. The quantitative data further demonstrated that generally, the expression of Aggrecan and Col II is higher on the nHA-terminated multilayers compared to the Col I-ended surfaces and is significantly higher than that of oHA and nCS-containing surfaces.

Overall, the chondrogenic differentiation of hUC-MSCs is considerably more pronounced on HA particularly on Col I/nHA than on nCS-containing surfaces, and the GAG-terminating surfaces generally promote more chondrogenesis than the Col I-terminated ones. These findings could be explained by the matrix composition and terminal layer in the multilayers. The incorporation of HA mimicking the cartilage niche has been found to enhance MSCs’ chondrogenesis in both 2D and 3D conditions *in vitro* (LeBoeuf et al., 1986; Knudson and Knudson, 1993), and increase the hyaline cartilaginous matrix formation *in vitro* (Allemann et al., 2001). Though nCS has been reported to promote chondrogenic-related gene expressions and cartilage-specific matrix production of MSCs (Varghese et al., 2008), it is worth noting that nCS also exists in osseous tissue, which is involved in the total bone metabolic process and has a stimulatory effect on osteogenesis of MSCs (Schneiders et al., 2012). When MSCs were cultured on nCS-containing materials rather than HA-incorporated ones, calcium phosphate deposition and osteogenic marker gene expression were significantly increased (Mathieu and Loboa, 2012; Wu et al., 2015). In addition, probably, hUC-MSCs are not only attached on the surface of the PEM but also penetrated into the underlying layers, there, more interpenetrated and fuzzy layers with significant more organized Col I fibrils underlying are found in the nCS-terminated PEM as detected by AFM. Compared to the cartilage-specific matrix GAG, Col I represents a major component of bone tissue, which is well known for its bioactivity in favor of osteogenesis (Kang et al., 2013; Zhao et al., 2016), and thus, the reduced chondrogenic differentiation capacity of hUC-MSCs on Col I-terminated surfaces is expected. However, compared to Col I/nHA, the chondrogenesis of hUC-MSCs was significantly lower on Col I/oHA. The previous study reported that increasing HA molecular weight (80–2000 kDa) increases the HA-induced chondrogenic effect on MSCs (Wu et al., 2018a). HA with higher molecular weight had the strongest chondrogenic effect on MSCs *in vitro* and exhibited pronounced cartilaginous tissue formation *in vivo*. However, after oxidation, the molecular weight of HA was greatly decreased, and thus, the HA-induced chondrogenic effect might also reduce.

#### 3.4.2 ERK/Sox9 and p38 pathways in the multilayer-dependent chondrogenic differentiation of hUC-MSCs

Since the chondrogenic differentiation of hUC-MSCs differs notably among different PEM, it is of great interest to understand the underlying mechanism. To address this question, we performed a WB assay to investigate the expression of proteins linked to the ERK/Sox9 and noncanonical TGF-β signal pathways. Sox9 is a cartilage-specific transcription factor that directly regulates the expression of cartilaginous-specific matrixes, such as aggrecan and Col II (Bell et al., 1997; I Sekiya et al., 2000). As predicted by the qPCR results, Sox9 expression was considerably higher in cells grown on Col I/nHA than in cells cultivated on the other PEM (see Figure 4 *).* According to a recent study, HA with a relatively large molecular weight (2000 kDa) had the strongest chondrogenic effect on MSCs *in vitro* and induced remarkable cartilaginous tissue formation *in vivo*, which was mediated through increased CD44 clustering and the ERK/Sox9 signaling pathway (Wu et al., 2018a). Meran and coworkers also found that enhanced HA-CD44 binding promotes late ERK activation (Soma Meran et al., 2011). It is evident that, compared to the other PEM, the protein levels of p-ERK1/2 and Sox9 are remarkably increased on Col I/nHA (see Figure 6A), showing that ERK activity may participate in Sox9-mediated chondrogenic differentiation of hUC-MSCs in response to HA stimulation in this bioactive PEM system. However, the protein levels of p-ERK1/2 and Sox9 are not so pronounced in the other HA-containing PEM (nHA/Col I, Col I/oHA, and oHA/Col I), since CD44 clustering is responsible for the upregulation of ERK phosphorylation, which positively regulates Sox9 expression and chondrogenic differentiation of MSCs. Notably, compared to the other HA-based PEM, CLSM showed that the clustering of CD44 was considerably more pronounced on Col I/nHA. In addition, TGF-β1 was added as a chondrogenic supplemental factor during the induction in our study. Previous reports demonstrated that CD44 can also function as a coreceptor of TGF-β, colocalizing with TGF receptors, and thus, facilitating regulation of both Smad-dependent and Smad-independent TGF-β1-mediated signal transduction (Ito et al., 2004; Soma Meran et al., 2008). TGF-β signaling is known to regulate many cellular fates, such as cell growth and differentiation, by activating either canonical (Smad-dependent) or noncanonical (nonSmad-dependent) pathways (van der Kraan et al., 2009; Zhang, 2009; Wu et al., 2015). According to a recent study, the p38 pathway, but not PI3K or ERK1/2, is required for TGF-β1-induced chondrogenic differentiation of mature MSCs in monolayer on plastic and in a 3D collagen-GAG scaffold (McMahon et al., 2008). As shown in Figure 6B, the protein levels of TGF-β and p-p38 are generally higher on HA-based multilayers than on nCS-containing multilayers. More specifically, the protein level of TGF-β and p-p38 are the highest on Col I/nHA which correlates also to the increased CD44 clustering and most pronounced chondrogenic differentiation, indicating the non-canonical TGF-β signaling may also involve in regulating the chondrogenic differentiation of hUC-MSCs on this PEM by activation of p38 pathway.

**FIGURE 7:**
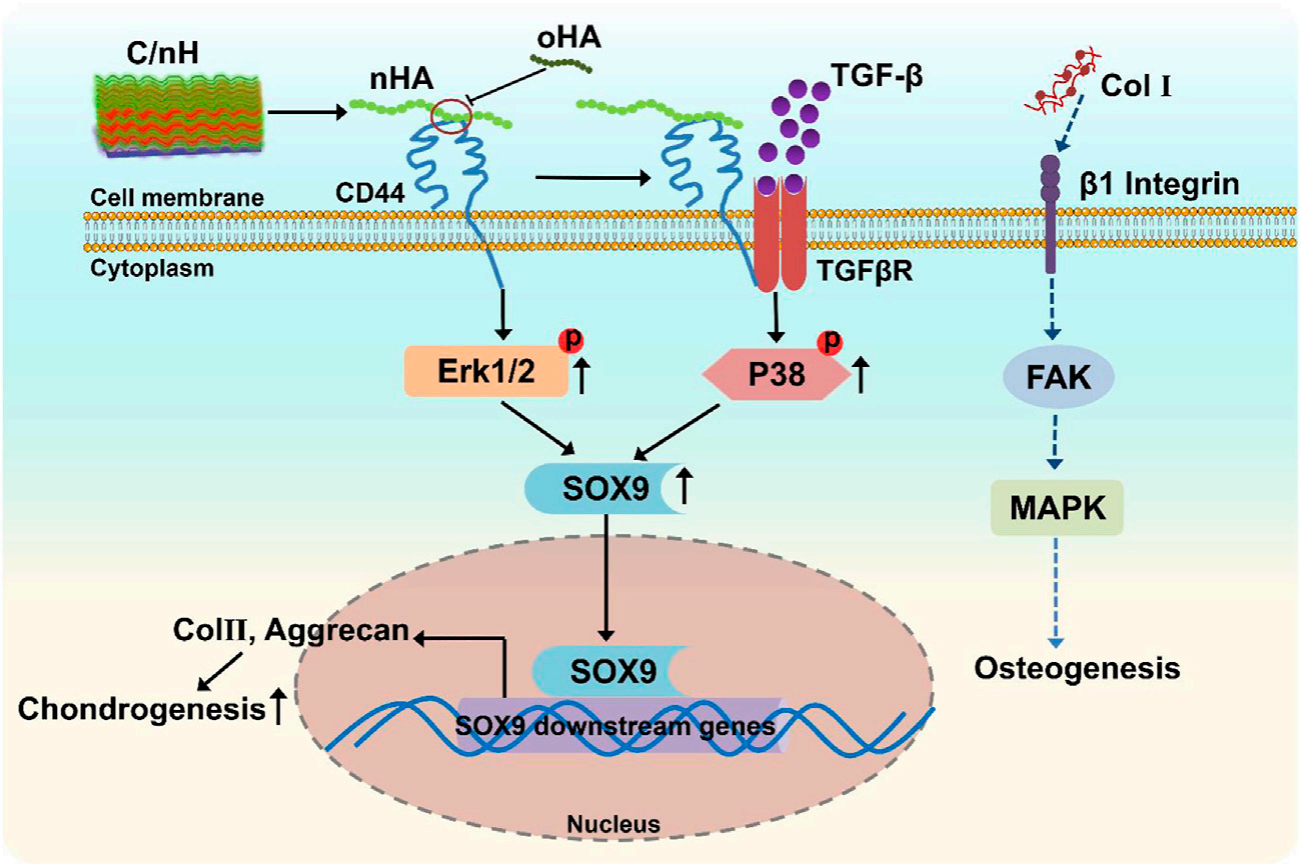
Scheme of the proposed mechanism of multilayer-promoted differentiation of MSC. nHA as component of Col I/nHA induce the clustering of CD44 which result in the activation of ERK/Sox9 and non-canonical TGF-β signaling pathways that are also related to induction of genes involved in chondrogenesis. Opposite to that oHA with lower molecular weight disrupts the clustering of CD44 which results in reduced signal transduction across these pathways resulting in lower chondrogenesis. Col I as further component of multilayers ligates and clusters β1 integrin which activates the focal adhesion kinase (FAK)-mitogen activated protein kinase (MAPK) signaling pathways that induces mitosis of cells but also promotes osteogenesis.

We deduced, due to the multitude of possible binding sites, the higher molecular weight nHA can effectively crosslink several CD44 receptors, and thus, stimulate CD44 clustering that activate the ERK/Sox9 pathway as well as the regulation of noncanonical TGF-β signaling through p38 pathway (Figure 7), which promoted chondrogenesis of MSCs. Nevertheless, the lower molecular weight oHA with reduced binding sites might disrupt CD44 clustering, and thus downregulated the chondrogenic differentiation of MSCs. In contrast, the presence of Col I fibrils on the Col I-terminated PEM is known to interact with β1 integrins and has been shown to trigger phosphorylation of focal adhesion kinase (FAK), downstream signaling by mitogen activated protein kinase (MAPK), which is correlated to the upregulation of osteogenic transcription factors, and thus promote osteogenesis.

## 4 Conclusion

In this study, we investigated the effect of a cartilage niche mimicking microenvironment fabricated by LBL technique, including changes in the molecular composition (type of GAG) and terminal component of PEM on the chondrogenic differentiation of hUC-MSCs and to further show the underlying mechanism. When chondrogenic medium supplements were used, HA-based, particularly nHA-terminated PEM with considerably less organized Col I fibrils, showed the higher bioactivity on promoting the chondrogenic differentiation of MSCs as in comparison to nCS-containing PEM, which is related to HA-CD44 interactions that activate the ERK/Sox9 pathway as well as the regulation of noncanonical TGF-β signaling through p38 pathway. However, the promoting effect of PEM was significantly decreased when nHA was replaced by oHA, which might cause by the decrease in molecular weight that reduced the clustering of CD44. These findings show for the first time the potential mechanism behind MSC differentiation in the presence of multilayers, which paved the way for the design of bioactive coatings mimicking tissue-specific matrixes applicable in tissue engineering and regenerative medicine. In addition, these cartilage niche mimicking multilayers based on HA and collagen can be used to upload TGF-β1 as shown in one of our recent publications using BMP-2 (Anouz et al., 2018) to overcome the lack of chondrogenic supplements *in vivo* and induce cartilage regeneration.

## Data Availability

The original contributions presented in the study are included in the article/Supplementary Material, further inquiries can be directed to the corresponding authors.
