# Supplementary material for: Chondrogenic differentiation of mesenchymal stem cells through cartilage matrix-inspired surface coatings
Source: Front Bioeng Biotechnol. 2022 Sep 29;10:991855. doi: 10.3389/fbioe.2022.991855 (PMC9557131; doi:10.3389/fbioe.2022.991855)
Supplement: Supplementary file 1 [file DataSheet1.doc]

**Chondrogenic Differentiation of Mesenchymal Stem Cells Through Cartilage Matrix-Inspired Surface Coatings**

Mingyan Zhao^1#*^, Xiang Gao^1#^, Jinsong Wei^2#^, Chenlin Tu^1,2^, Hong Zheng^2^, Kaipeng Jing^3^, Jiaqi Chu^1^, Wei Ye^4^, Thomas Groth^5*^

^1^Stem Cell Research and Cellular Therapy Center, Affiliated Hospital of Guangdong Medical University, Zhanjiang 524001, China

^2^Department of Spinal Surgery, Affiliated Hospital of Guangdong Medical University, Zhanjiang 524001, China

^3^Key Laboratory of Prevention and Management of Chronic Kidney Disease of Zhanjiang City, Institute of Nephrology, Affiliated Hospital of Guangdong Medical University, Zhanjiang 524001 China

^4^Department of Obstetrics and Gynecology, Affiliated Hospital of Guangdong Medical University, Zhanjiang 524001 China

^5^Department Biomedical Materials, Institute of Pharmacy, Martin Luther University Halle Wittenberg, Halle (Saale) 06120, Germany

^#^They should be regarded as the co-first authors.

Corresponding authors:

Mingyan Zhao: mingyan.zhao@gdmu.edu.cn; phone: +86 (0759) 2386010; fax: +86 (0759) 2231754

Thomas Groth: thomas.groth@pharmazie.uni-halle.de; phone: +49 (0345) 55 28460; fax: +49 (0345) 5527379

1. **Materials and Methods**
   1. **Molecular Weight Detection**

The molecular weight of oxidized hyaluronan (oHA) was determined using asymmetrical flow field-flow fractionation (A4F) with a Dawn EOS detector (Wyatt Technology Corporation, Goleta, USA) and a refractive index (RI)-detector (Shodex RI–101, Showa Denko Europe GmbH, Munich, Germany). Samples were measured in sodium chloride (0.05 M, Sigma, Steinheim, Germany) supplemented with 0.02% sodium azide (sigma) (w/v). Data were calculated with an Astra software (Wyatt Technology Corporation) to obtain the molecular weights.

**1.2 Surface Wettability and Surface Potential Measurements**

The wettability of various polyelectrolyte multilayers (PEM) surfaces on glass coverslips was investigated by static water contact angle (WCA) measurements with fresh ultrapure water using an OCA 15+ device from Dataphysics GmbH (Filderstadt, Germany). Each sample was measured in triplicate using the sessile drop method. One microliter of ultrapure water was dispensed by a motorized syringe onto the sample (five droplets per sample), and WCA values were determined using the ellipse fitting method[1].

The zeta potential of various PEM was measured using the SurPASS device by Anton Paar (Graz, Austria). Two identical multilayer-coated glass coverslips (size 10×20 mm^2^) were fixed on stamps with double-sided tape and placed in the SurPASS flow cell in opposing directions. The gap of the flow cell was adjusted to obtain a flow rate of 100-150 mL min^−1^ at a maximum pressure of 300 mbar. A solution of 1 mM KCl was used as a model electrolyte, and 0.1 M HCl was used for pH titration. The pH of the model electrolyte was adjusted to 10.5 using 1 M NaOH before starting the measurement, followed by an automated titration from pH 10.5 to 2.25. The electrolyte solution was purged with nitrogen throughout the measurement. Each measurement was performed three times.

**1.3 Cell Adhesion Investigation**

Before cell seeding, the plain or multilayer-coated glass coverslips were sterilized by UV irradiation (for 30 min on each side) and then placed in 24-well tissue culture plates (NEST, Wuxi, China). Serum-free suspensions of hUC-MSCs at a concentration of 10,000 cells mL^−1^ were prepared and seeded on plain or multilayer-coated samples. After incubating for 4 h, samples were rinsed once with PBS and the cells were stained with crystal violet solution (Solarbio, Beijing, China) for 30 min. Thereafter, the samples were carefully rinsed with distilled water and dried in air. Images were taken using an inverted fluorescence microscope (DMI 3000B, Leica, Wetzlar, Germany). The number of adherent cells and their morphology, such as mean cell area and aspect ratio, were analyzed using ImageJ from five images per sample (three for each type of multilayer) (NIH, USA).

**1.4 Cell Migration and Growth Investigations**

The effect of different PEM on the migration of hUC-MSCs was determined using a scratch test as previously described[2]. hUC-MSCs were seeded on multilayer-coated 6-well plates (NEST) at a concentration of 2.5×10^5^ cells per well and grown to confluence. The cells seeded on a plain plate were used as a control. Next, a clear scratch was carefully made through the cell monolayer using a P200 pipette tip. Cells were washed twice with PBS and grown in L-DMEM supplemented with 10% FBS and 1% penicillin-streptomycin at 37℃ in a humidified environment containing 5% CO_2_. Subsequently, the migration of cells into the scratched area was monitored by taking pictures at predetermined time. Quantitative results of cell migration were analyzed from four images per sample based on the following calculation:

(1)

Where t_0_ denotes the time immediately (0 h) after making the scratch, whereas t_s_ represents the defined time (6 and 12 h, respectively) after making the scratch.

The proliferation of hUC-MSCs plated on the different PEM was determined using an MTS cell proliferation kit (Promega, Madison, WI) after 1, 2, and 3 days of incubation. This was accomplished by aspirating the old medium and washing samples once with sterile PBS. Next, 300 μL of L-DMEM supplemented with MTS test reagent (a ratio of 10:1) was added to each well and incubated for an additional 2 h. Following that, the absorbance of MTS reagent was measured at 492 nm using a plate reader (Thermo Fisher, USA) to determine the number of live cells.

1. **Results and discussion**

**2.1 Molecular Weight of Oxidized Hyaluronan (oHA)**

As shown in **Table S1**, the oxidization leads to a large decrease in molecular weight, which decreased from 1300 kDa to 55 kDa. Periodate oxidation not only breaks the vicinal glycols in polysaccharides to form their oxidized derivatives, but also hydrolyzes the glycosidic bond, which causes a decrease of molecular weight of HA.

**Table S1:** Molecular weight of native (nHA) and oxidized hyaluronan (oHA).

| Samples | Molecular Weight (kDa) |
| --- | --- |
| nHA | 1300 |
| oHA | 5.5 |

**2.2 Measurements of Wetting Properties and Zeta Potentials**

Static WCA measurements were performed to monitor the assembly and wettability of the PEM. The trend in WCA values after the deposition of each layer and the WCA values of the outermost layer of each PEM are shown in **Figures S1** (**A and B**). An alternating trend of WCA values was observed for all the PEM, where WCA values were higher when Col I was adsorbed, while they were lower with the GAG (nHA, oHA, or nCS) adsorption. Such alternating WCA differences after each adsorbed layer reflect the varying molecular composition of the terminating layer, and the higher differences indicate the formation of more separated multilayers [3, 4], since HA and nCS are both highly hydrophilic polysaccharides, while Col I is more hydrophobic [5]. It is interesting to note that the WCA trend for HA-containing PEM strongly differed from that of nCS-based PEM after the fifth layer. There, the WCA increased to a lesser degree after the deposition of Col I but decreased to a higher extent after subsequent nHA or oHA adsorption, showing that the WCA of the following nHA or oHA layer was similar to the previous nHA or oHA layers. In contrast, the WCA of a nCS-based PEM increased as the layer number increased, with the WCA of the subsequent nCS layer being higher than that of the previous nCS layer. These findings provide further evidence showing that GAG dominate in HA-containing PEM, while Col I produces considerably higher mass in nCS-based PEM. In addition, it is evident (see **Figure S1 B**) that the WCA value of the nCS-containing PEM is significantly higher than that of the HA-based PEM (both nHA and oHA), while Col I terminal layers is always slightly higher than that of the GAG-terminated layer, though no significant difference was found.

Streaming potential measurements were performed to detect the surface zeta potential after multilayer formation as a function of pH value. The results are shown in **Figure S1C** and **D**. The zeta potential between the different PEM were comparable and no significant difference was observed when the pH value was lower than 5, as shown in **Figure S1C**. However, as pH steadily increased, nHA-based PEM demonstrated lower potentials than nCS-based ones, particularly in the alkaline regions. The higher zeta-potential values for the nCS-based PEM show that the polycation Col I plays a dominant role in these PEM, as demonstrated by QCM, AFM, and WCA measurements. In addition, a lower zeta potential was found on nHA-based PEM at pH values >6.5 when compared to oHA-containing PEM with a higher wettability, which could show a loss of Col I after exposing the multilayers to electrolyte solutions with higher pH values during the measurements. Moreover, as shown in **Figure S1D**, the HA-terminated multilayers have a considerably lower surface zeta potential than the Col I-terminated multilayers, whereas the nCS and Col I-terminated multilayers show no significant difference. Presumably, there are more separated multilayers in the HA-based PEM due to the larger size of the polyanion nHA or oHA dominating the terminal layer regarding surface charge and more interpenetrated, fuzzy multilayers formed by nCS-based PEM. In addition, the QCM investigation indicated that nCS diffused more readily into the underlying layers than HA, which is also related to the smaller size of nCS and its higher charge density. The same phenomenon has been found in other PEM systems[4, 6].


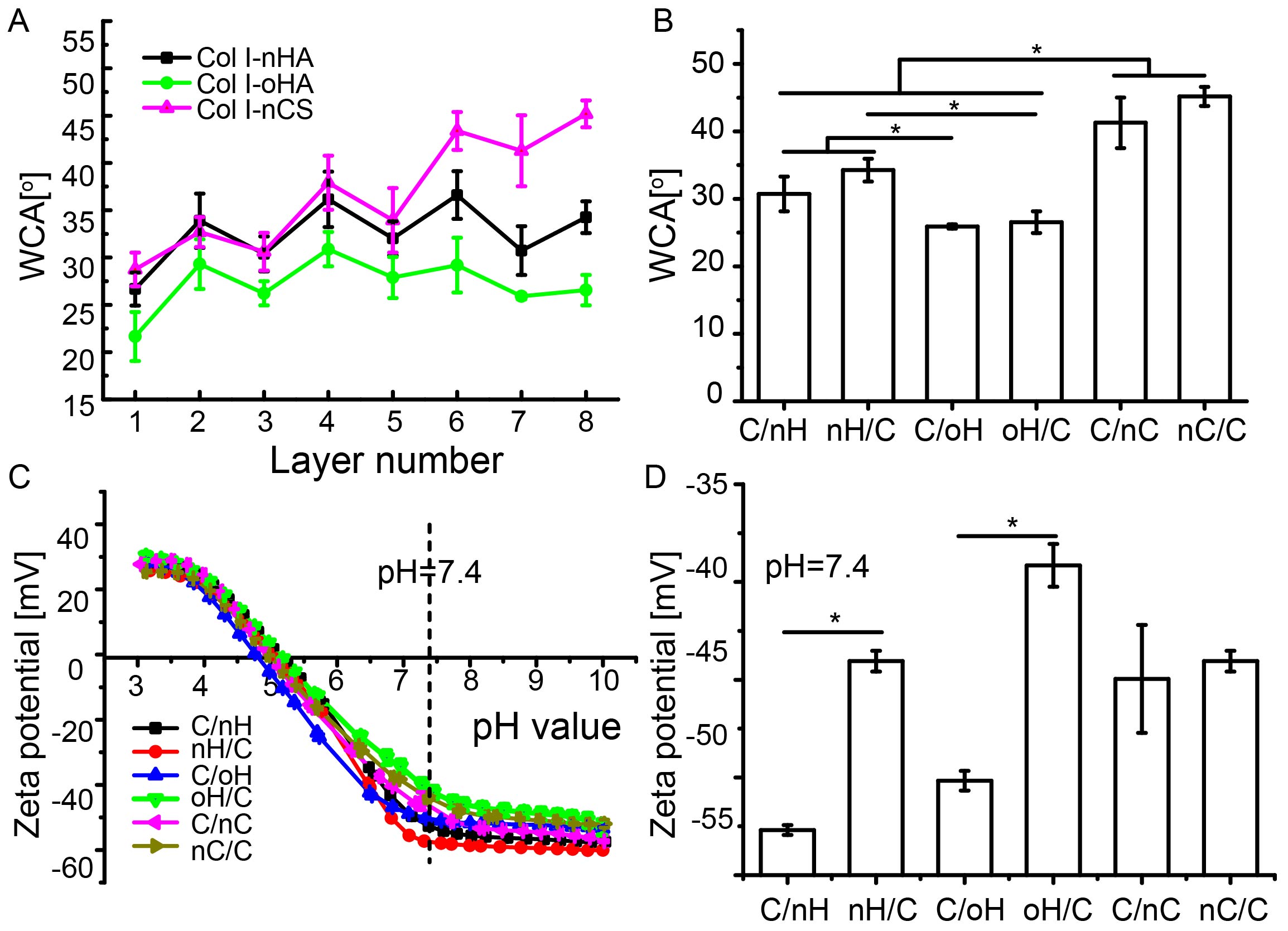


**Figure S1.** Surface wettability and surface zeta-potentials of different PEM. (A) Static WCA measurements during multilayer formation up to eight layers; (B) The WCA value of outermost Col I and polyanion terminal layers for the different multilayer systems; (C) Zeta-potential measurements of outermost Col I and polyanion terminal layers as a function of pH value, and (D) Zeta-potentials measured at pH 7.4 for the different PEM.

**2.3 Initial interactions of hUC-MSCs with Polyelectrolyte Multilayers (PEM)**

Cell count and morphology, such as cell area and aspect ratio, were quantified using images of cells stained with crystal violet, as shown in **Figure S2**. Although no significant difference in cell spreading was found among the different PEM, the cell area was always higher while the aspect ratio was lower on the Col I/nHA and nCS/Col I multilayers, indicating improved cell spreading and elongated cell morphology on these PEM. Moreover, the cell adhesion and spreading are also slightly improved on the nCS-containing and Col I-terminated multilayers when compared to the HA (both nHA and oHA)-based ones, which could be explained by the presence of more Col I in nCS/Col I multilayers as proven by QCM, WCA, zeta potential measurements (**Figure 1** and **Figure S1**), and AFM (**Figure 2**). The higher Col I deposition was also accompanied by a distinguished and well-interconnected fibrillar structure of Col I in the nCS/Col I multilayers, which resulted in a moderately wettable surface (**Figure S1**). Previous studies revealed that the presence of collagen fibrils enhanced the adhesion of a variety of cells via an integrin-mediated mechanism[7], particularly via the integrin α2β1 supporting the adhesion of cells on substrata containing fibrillized Col I[8]. However, the moderately wettable surface of nCS/Col I may further improve cell spreading, as compared to highly hydrophobic or hydrophilic surfaces, cells prefer to attach to moderately wettable surfaces [46]. Nevertheless, the improved adhesion and spreading of hUC-MSCs on Col I/nHA cannot be simply explained by the bioactivity of adsorbed Col I or surface wettability. We hypothesize that this is related to the clustering of hyaladherin CD44, the main HA receptor[9], because adhesion-receptor ligation is important in connecting the ECM with the cell cytoskeleton and signaling complexes[10]. Indeed, as shown in **Figure 3A and Figure S2D**, CD44 was considerably more expressed and organized in hUC-MSCs adhering on Col I/nHA surfaces.


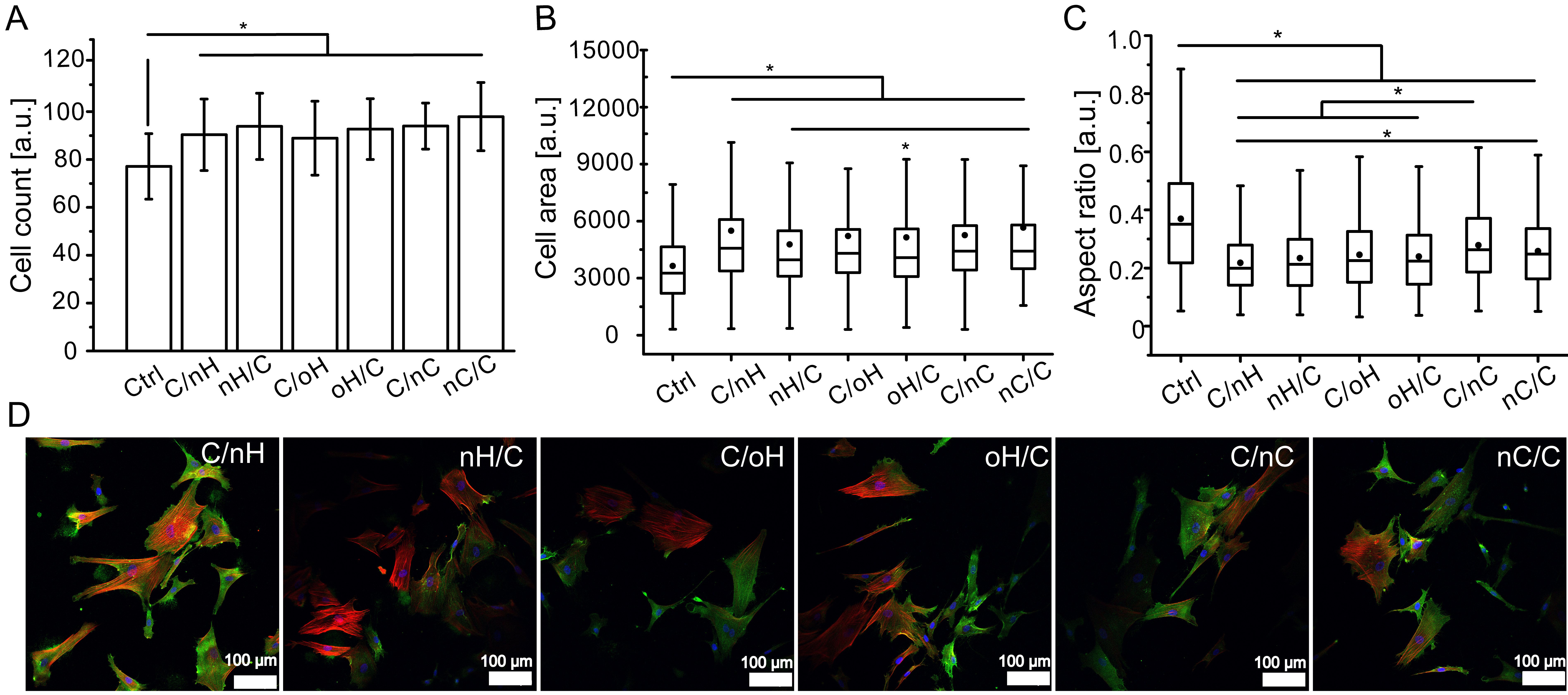


**Figure S2**. Adhesion (A), size (B), morphology (C, Box-whisker diagram shows the 25th and 75th percentile and mean values as small black squares), CLSM (D) with staining of CD44 (green), actin (red) and nuclei (blue) of hUC-MSCs after 4 hours incubation on the different multilayers-coated surfaces. [PEM were prepared with terminal layer of nHA, oHA and nCS (C/nH, C/oH, C/nC) or outermost layer of Col I (nH/C, oH/C, nC/C). Cells were seeded in serum free medium for 4 hours].

Cell migration is a prerequisite in regeneration of all tissues. To initiate any regeneration based on MSC activity, the cells must first be recruited to the site of damage[11]. Subsequently, the adhesion of MSCs to a local matrix followed by activation and extensive proliferation to provide the necessary high numbers of chondroprogenitor cells to regenerate new tissue is required[11]. In this context, the effects of molecular composition and terminal layer on the migration activity and proliferation of hUC-MSCs were studied using a scrape injury assay and an MTS assay, respectively. The representative images of cell migration (**Figure S3A**) and the quantitative results (**Figure S3C**) clearly demonstrate that cell migration is improved on the Col I-terminated surfaces, especially nCS/Col I surfaces, after 6 h of culture, though no significant difference was found. Nevertheless, this is not the case for the nHA-containing multilayers, where the cell migration activity was even slightly higher for Col I/nHA compared with nHA/Col I. Quantitative data of cell growth or viable cells after the different culture periods were determined by the MTS assay, as shown in **Figure S3C**. In correspondence to that of adhesion and migration studies, there was no significant difference in the quantity of metabolically active cells on different PEM after one day of culturing, while a significantly lower quantity of metabolically active cells was found on the GAG-terminated multilayers compared to the respective Col I-terminated ones at longer culture times. However, no obvious difference was observed between the Col I/nHA and nHA/Col I. Overall, the migration and growth of hUC-MSCs were obviously improved on Col I and nHA-terminated multilayers. The presence of outermost Col I fibrils is known to promote the adhesion of a variety of cells via an β1 integrin-mediated mechanism, which triggers signal transduction via the mitogen activated protein kinase pathways, and thus, promotes cell migration and growth[8, 12]. In addition, it is well known that HA interactions with cell surface receptors, primarily CD44 and HA-mediated motility (RHAMM), are associated with the activation of several signaling pathways [e.g., focal adhesion kinase, extracellular signal-regulated protein kinase (ERK), and protein kinase B (AKT)] that regulate cell adhesion, migration, and proliferation[13, 14]. The nHA-terminated multilayers obviously increased CD44 clustering, and thus, promoted the migration and proliferation of hUC-MSCs.


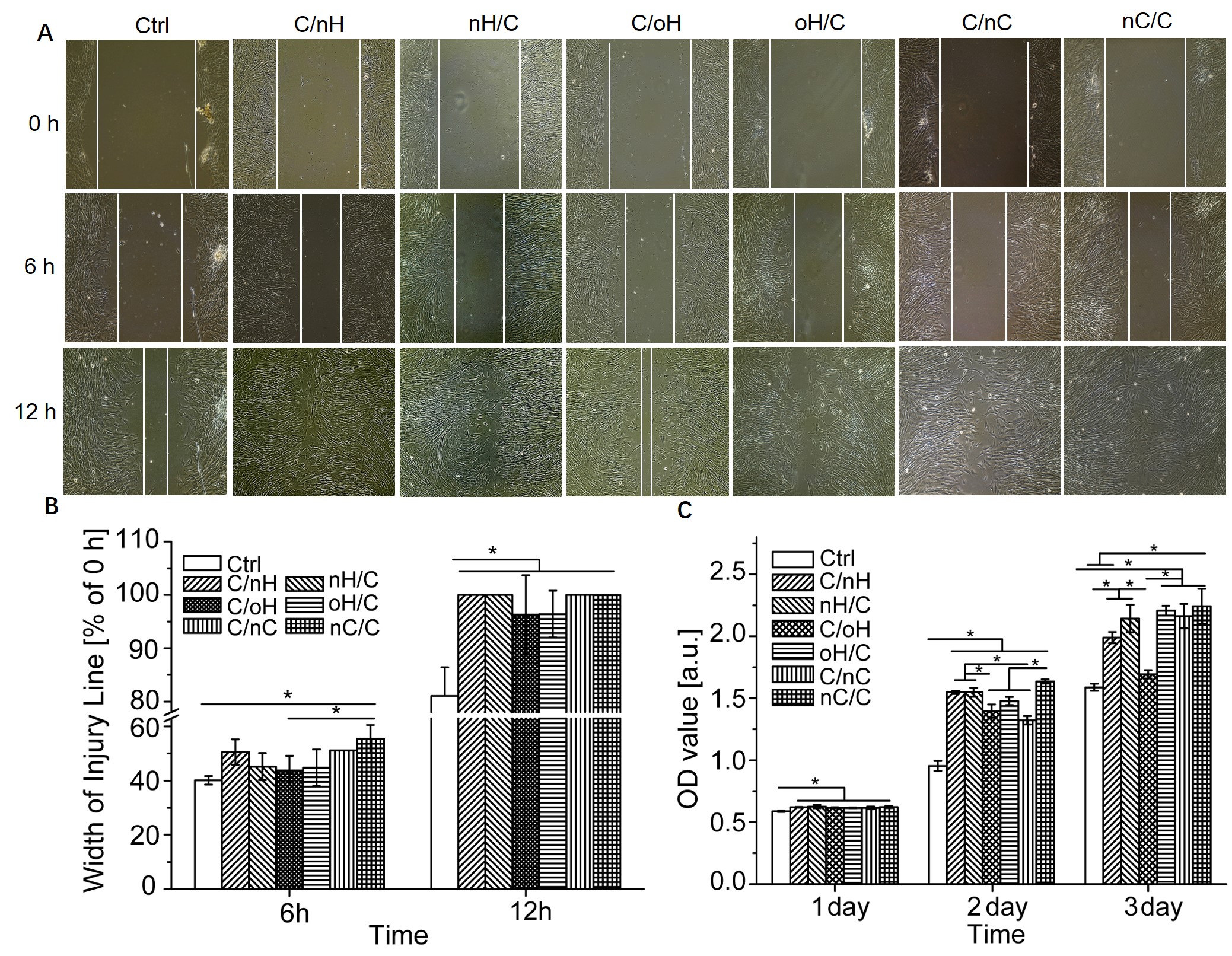


**Figure S3.** The migration activity and proliferation of hUC-MSCs grown on different surfaces. (A) Representative images of cell migration at different time point (×100); (B) The quantitative data of cell migration activity in control group and each multilayer surfaces. Cell migration was determined by scratch test, and the width of injury was monitored by photographing at 0, 6, and 12 hours. Cell migration (%) was quantified by calculating the injury width; (C) Measurement of metabolic active cells by MTS assay during the predetermined culture time (1, 2 and 3 days respectively). Results are presented as means ± SD of three independent experiments. The multilayers are the same as described in **Figure S2**.


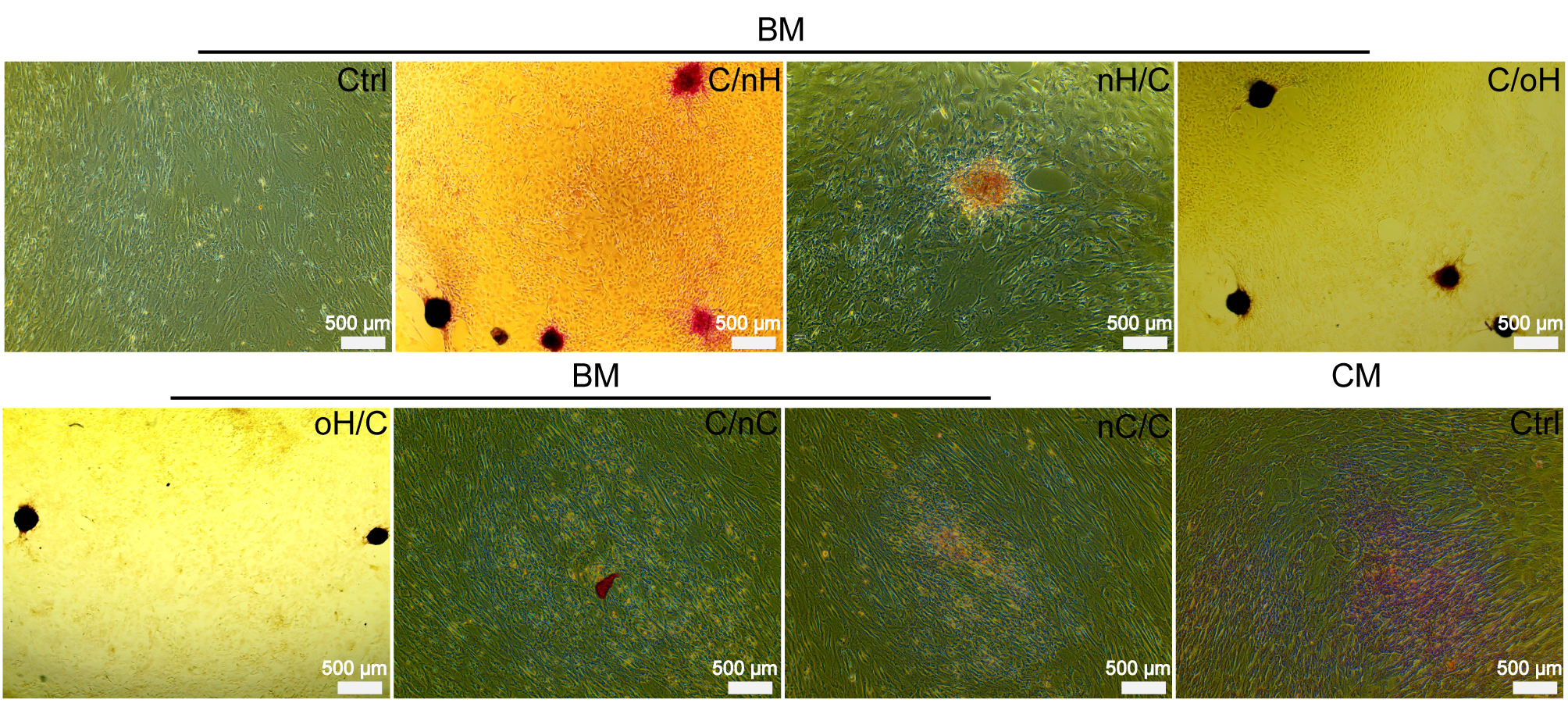


**Figure S4**. Safranin O staining for acidic glycosaminoglycans (GAGs) in hUC-MSCs placed on the different PEM treated with basial medium (BM) or chondrogenic differentiation medium (CM) (×100). The multilayers are the same as described in **Figure S2**.

**References:**

1. Zhao M, Li L, Zhou C, Heyroth F, Fuhrmann B, Maeder K, Groth T: **Improved Stability and Cell Response by Intrinsic Cross-Linking of Multilayers from Collagen I and Oxidized Glycosaminoglycans**. *Biomacromolecules* 2014, **15**(11):4272-4280.

2. Zhao M, Li P, Xu H, Pan Q, Zeng R, Ma X, Li Z, Lin H: **Dexamethasone-Activated MSCs Release MVs for Stimulating Osteogenic Response**. *Stem Cells International* 2018, **2018**:7231739.

3. Aggarwal N, Groth T: **Multilayer films by blending heparin with semisynthetic cellulose sulfates: Physico–chemical characterization and cell responses**. *Journal of Biomedical Materials Research Part A* 2014, **102**(12):4224-4233.

4. Aggarwal N, Altgärde N, Svedhem S, Zhang K, Fischer S, Groth T: **Effect of Molecular Composition of Heparin and Cellulose Sulfate on Multilayer Formation and Cell Response**. *Langmuir* 2013, **29**(45):13853-13864.

5. Taraballi F, Zanini S, Lupo C, Panseri S, Cunha C, Riccardi C, Marcacci M, Campione M, Cipolla L: **Amino and carboxyl plasma functionalization of collagen films for tissue engineering applications**. *Journal of Colloid and Interface Science* 2013, **394**:590-597.

6. Niepel MS, Peschel D, Sisquella X, Planell JA, Groth T: **pH-dependent modulation of fibroblast adhesion on multilayers composed of poly(ethylene imine) and heparin**. *Biomaterials* 2009, **30**(28):4939-4947.

7. N.M. Coelho CG-G, J.A. Planell, M. Salmeron-Sanchez, G. Altankov: **Different assembly of type IV collagen on hydrophilic and hydrophobic substrata alters endothelial cells interaction**. *Eur Cell Mater* 2010, **19**:262-272.

8. Zhao M, Altankov G, Grabiec U, Bennett M, Salmeron-Sanchez M, Dehghani F, Groth T: **Molecular composition of GAG-collagen I multilayers affects remodeling of terminal layers and osteogenic differentiation of adipose-derived stem cells**. *Acta Biomaterialia* 2016, **41**:86-99.

9. Ouasti S, Kingham PJ, Terenghi G, Tirelli N: **The CD44/integrins interplay and the significance of receptor binding and re-presentation in the uptake of RGD-functionalized hyaluronic acid**. *Biomaterials* 2012, **33**(4):1120-1134.

10. McEver RP, Luscinskas FW: **Chapter 12 - Cell Adhesion**. In: *Hematology (Seventh Edition).* Edited by Hoffman R, Benz EJ, Silberstein LE, Heslop HE, Weitz JI, Anastasi J, Salama ME, Abutalib SA: Elsevier; 2018: 127-134.

11. Richter W: **Mesenchymal stem cells and cartilage in situ regeneration**. *Journal of Internal Medicine* 2009, **266**(4):390-405.

12. Hynes RO: **Integrins: Versatility, modulation, and signaling in cell adhesion**. *Cell* 1992, **69**(1):11-25.

13. Carvalho AM, Soares da Costa D, Paulo PMR, Reis RL, Pashkuleva I: **Co-localization and crosstalk between CD44 and RHAMM depend on hyaluronan presentation**. *Acta Biomaterialia* 2021, **119**:114-124.

14. Akiyama Y, Jung S, Salhia B, Lee S, Hubbard S, Taylor M, Mainprize T, Akaishi K, van Furth W, Rutka JT: **Hyaluronate Receptors Mediating Glioma Cell Migration and Proliferation**. *Journal of Neuro-Oncology* 2001, **53**(2):115-127.
